# Using geographic rescue time contours, point-of-care strategies, and spatial care paths to prepare island communities for global warming, rising oceans, and weather disasters

**DOI:** 10.1186/s12942-023-00359-y

**Published:** 2023-12-20

**Authors:** Gerald J. Kost, Anna K. Füzéry, Louie Kim R. Caratao, Samantha Tinsay, Amanullah Zadran, Adrian P. Ybañez

**Affiliations:** 1 Fulbright Scholar 2020-2022, ASEAN Program, Point-of-Care Testing Center for Teaching and Research (POCT•CTR), Pathology and Laboratory Medicine, School of Medicine, University of California, Davis, CA 95616 USA; 2 Department of Laboratory Medicine and Pathology, University of Alberta, Edmonton, Alberta Canada; 3 Cebu Technological University, Cebu, Philippines; 4 Municipality of Bantayan, Bantayan-Santa Fe-Madridejos Primary Care Provider Network, Cebu, Philippines; 5 POCT·CTR, Public Health Sciences, School of Medicine, University of California, Davis, USA; 6 Institute for Molecular Genetics, Parasitology, and Vector-Borne Diseases, and College of Veterinary Medicine, Cebu Technological University, Cebu, Philippines

**Keywords:** Climate change, Geographic rescue time contours, Global warming, Island communities, Medical emergencies, Point-of-care strategies, Rising oceans, Spatial care paths, Spatial injustice, Weather disasters

## Abstract

**Objectives:**

To perform geographic contour analysis of sea and land ambulance rescue times in an archipelago subject to super typhoons; to design point-of-care testing strategies for medical emergencies and weather disasters made more intense by global warming and rising oceans; and to assess needs for prehospital testing on spatial care paths that accelerate decision making, increase efficiency, improve outcomes, and enhance standards of care in island nations.

**Methods:**

We performed needs assessments, inspected healthcare facilities, and collected ambulance rescue times from professionals in the Bantayan Archipelago, Philippines. We mapped sea/land ambulance rescue routes and time contours. To reveal gaps, we statistically compared the fastest and slowest patient rescue times from islands/islets and barangays to the District Hospital on Bantayan Island. We developed spatial care paths (the fastest routes to care) for acute myocardial infarction, community care, and infectious diseases. We generated a compendium of prehospital diagnostic testing and integrated outcomes evidence, diagnostic needs, and public health goals to recommend point-of-care strategies that build geographic health resilience.

**Results:**

We observed limited access to COVID-19 assays, absence of blood gas/pH testing for critical care support, and spatial gaps in land and airborne rescues that worsened during inclement weather and sea swells. Mean paired differences (slowest-fastest) in ambulance rescue times to the District Hospital for both islands and barangays were significant (P < 0.0001). Spatial care path analysis showed where point-of-care cardiac troponin testing should be implemented for expedited care of acute myocardial infarction. Geospatial strengths comprised distributed primary care that can be facilitated by point-of-care testing, logical interisland transfers for which decision making and triage could be accelerated with onboard diagnostics, and healthcare networks amenable to medical advances in prehospital testing that accelerate treatment.

**Conclusions:**

Point-of-care testing should be positioned upstream close to homes and island populations that have prolonged rescue time contours. Geospatially optimized point-of-need diagnostics and distributed prehospital testing have high potential to improve outcomes. These improvements will potentially decrease disparities in mortality among archipelago versus urban dwellers, help improve island public health, and enhance resilience for increasingly adverse and frequent climate change weather disasters that impact vulnerable coastal areas. [350 words].

**Supplementary Information:**

The online version contains supplementary material available at 10.1186/s12942-023-00359-y.

## Objectives

This research focuses on climate change and strategic preparation for its adverse effects. The objectives were to perform geographic contour analysis of best- and worst-case sea/land ambulance rescue times in an isolated archipelago subject to super typhoons and then to create needs-based point-of-care testing (POCT) strategies that accelerate decision making, increase efficiency, and improve outcomes in the healthcare small-world network faced with global warming, rising oceans, inundation, and increasingly, weather disasters. A compendium of prehospital testing (Additional file 1) shows how care can be accelerated and enhanced by positioning diagnostic tests upstream on spatial care paths, the fastest routes to diagnostic portals, decision making, and medical assistance.

## Background

Global warming intensifies storm severity, creating more frequent super typhoons greater in size and scope of damage. The Earth is losing 1.2 trillion tons of ice per year, and each centimeter of sea level rise forces one million people to move from low-lying homelands [1, 2]. Ocean levels are projected to rise 2.2 m (7 feet, 3 inches) by 2150 if climate change is not abated [3]. There are 900,000 islands in the world of which 16,000 are inhabited by 730,000,000 people, 11% of the world’s population.

Increasingly extreme weather events have caused a surge in natural disasters that disproportionately impact poorer countries. In the past 50 years, these hazards have accounted for 50% of all disasters, 45% of reported deaths, and 74% of reported economic losses. There have been more than 11,000 disasters globally, over two million deaths, and $3.64 trillion in economic losses with more than 91% of deaths occurring in developing countries [4].

Small increases in ocean levels from global warming magnify typhoon surges, flooding, and inundation [3]. Notably, storm surges can cause more damage and loss of life than the high velocity winds encountered in a typhoon. Storm intensity is increasing, and poor planning can adversely impact ambulance transport, critical care, and medical outcomes, as well as responses to everyday emergencies.

The Philippines has 7641 islands with 2000 of the islands inhabited [5].The island nation lies in the “typhon alley” of the Pacific, an extremely vulnerable location. In December 2021, the Visayas Islands, a group of seven large and hundreds of smaller islands situated among the Visayan, Samar, and Camotes Seas, were hit by the devastating super typhoon Odette [6]. Odette caused a major disaster, loss of 410 lives, and over $1 billion in damage.

Archipelagos are perpetually at risk. People will be forced to move from coastal areas and concentrate at higher density on larger or higher islands. We selected the remote Bantayan Archipelago located in Cebu Province, Visayas, for detailed geographic contour mapping of the fastest and slowest ambulance rescue times and the introduction of POCT strategies that can help meet urgent needs, improve community resilience, and prepare for an unpredictable future.

## Methods

### Definitions

Point-of-care testing is medical testing at or near the site of patient care [7]. A diagnostic portal is the geographic entry point from home to hospital where the patient self-tests or first seeks, obtains, or is administered diagnostic testing to rule in or rule out a medical condition and then expeditiously receives care [8].

A small-world network (SWN) represents the integrated system of emergency medical services (EMS), rescue routes, healthcare sites, diagnostic resources, medical specialists, and telecommunications that, among other features, assure wellness for a community in the context of its local geography, topography, and culture [9, 10]. Rescue time is the total time from the victim or patient encounter at the rescue site to arrival at the district hospital on Bantayan Island.

The physical SWN [i.e., SWN(p)] can be transformed into a time domain network, *SWN(t),* that anticipates the dynamics of responses and rescues [9, 10]. We temporally transformed the Bantayan Archipelago SWN by plotting ambulance rescue time contours based on raw data collected from local professionals and the Bantayan public health team led by coauthor S.T., who directly oversees public health in the archipelago.

A spatial care path is the fastest route the patient takes through the healthcare SWN(p) to medical care facilitated by point-of-care (POC) screening, testing, and monitoring to merge prevention and intervention and enhance standards of care [11–16]. Spatial care paths can be designed for unusual topologies such as those found in island communities. They may include home self-testing and mobile testing. Understanding spatial care path designs allows one to functionally optimize POCT geographic placements at diagnostic portals on the routes from homes to the sites of medical treatment.

### Needs assessment

We assessed needs scientifically based on methods [17–20] codified by multicenter teams from the NIH National Institute of Biomedical Imaging and Bioengineering Point-of-care Technologies Centers and Research Network [21–23]. We generated a collection tool for needs assessment data (Additional file 2), which was synthesized for use in the Philippines from prior POCT needs assessment questionnaires used in Cambodia [24, 25], Taiwan [26], Thailand [27–33], Vietnam [16, 34–36] and southern Thai provinces following the Andaman Sea Earthquake and Tsunami [30, 32, 33].

During visits to Bantayan Archipelago and Cebu Island healthcare facilities, we recorded information regarding access to diagnostic tests and ambulance services obtained from onsite discussions, facility inspections, and records provided by EMS, hospital leadership, and clinical laboratory staff, who gave us lists of diagnostic tests performed. We provided copies of Additional file 2 for the district hospital and rural health units, so personnel could answer pertinent questions and send responses to the research team at Cebu Technological University. We continued calling and sending email until the response rate for documents returned was 100%. Anecdotal information and Additional file 2 responses were integrated to produce the summary tables presented below in results.

Needs assessment topics comprised (a) facility overview and demographics, (b) POCT, (c) patient transport and critical care access, (d) clinical laboratory, (e) medical problem solving in the community, and (e) summary of field observations. We documented global positioning system coordinates to facilitate accurate mapping of healthcare resources to a nautical grid with background longitude and latitude [37].

We focused on sea and land ambulance rescue, emergency medicine, critical care testing (e.g., blood gases, pH, electrolytes, and ionized calcium), cardiac biomarkers, Coronavirus infectious disease 2019 (COVID-19) testing, POCT, prehospital diagnosis, clinical laboratories, and the organization of resources in the healthcare SWN.

We collated demographic facts about each medical site and laboratory, such as the number of hospital beds, professional services, status of critical care units, diagnostic test clusters, and the impact of patients diagnosed with COVID-19, acute myocardial infarction, and other urgent conditions on healthcare resources.

We worked with local officials and engineering staff to detail transportation links between outlying islands, Bantayan Island, and referral centers in Cebu City. We generated contour maps of minimum and maximum ambulance rescue times on land and sea; identified locations of ports, helipads, and airports; asked personnel about the value of POCT; and determined current capabilities versus future needs.

We met with hospital directors and administrators, clinical chiefs, physicians, nurses, EMS personnel, land and sea ambulance operators, laboratory technologists, clinicians, and academic advisors. The categories and number of participants comprised: Administrative Officer, 11; Administrative Head, 1; Bantayan Island Resident, 5; Chief/Head of DRRMO (Disaster Risk Reduction and Management Office), 5; Healthcare Company Chief Executive Officer, 1; Director/Chief of Hospital, 4; DRRMO/Rescue/EMS Officer 14; Emergency Medicine Department Head, 1; Engineer, 1; Ethics Head, 1; Head of Facility, 2; and Head of Public Health, 1.

Further: Human Resource Officer, 1; Laboratory Assistant, 7; Land Ambulance Driver, 4; Lineman (assigned in Bantayan Islets), 1; Manager, 1; Medical Laboratory Technologist, 16; Midwife and Birthing Care Staff, 3; Municipal Environment and Natural Resources Officer, 1; Municipal Health Officer, 5; Municipal Office Personnel, 1; Nurse, 3; Office Staff (CTU), 2; Operations Head, 1; Pathology Department Head, 1; POC Coordinator, 1; Public Health Nurse, 1; Pumpboat/Sandboat Operator, 4; Radiology Technologist, 1; Sea Ambulance Operator, 2; State University College President, 1; University Researcher, 1; University Vice-President of Research and Development, 1; Van drivers, 2—Total participants, 108.

### Geospatial analysis

We created ambulance rescue time contour maps and statistically compared the fastest (best-case) and slowest (worst-case) times for emergency sea and land victim and patient conveyance to the District Hospital on Bantayan Island in order to understand where to position POCT. Geospatial analyses were based on raw data provided by local professionals in the Bantayan Archipelago, which does not have helicopter or fixed wing ambulance aircraft readily available. We documented the topographical and temporal characteristics of medical delivery systems within and between islands in the archipelago.

DRRMO Chiefs in the three municipalities of the Bantayan Archipelago completed raw data spreadsheets listing the slowest and fastest ambulance rescue times to Bantayan District Hospital. These quantitative collations covered barangay, island, and islet ambulance time intervals. Estimates of ambulance speeds were applied for some Santa Fe barangays that lacked details. We used OpenStreetMap and Google Maps to confirm distances.

DRRMO leadership stated that ambulances traveled on main roads to avoid hazards potentially encountered on small connectors and shortcuts. Transfer intervals were not linearly distributed across the highway geography because of ambulance passage through town zones, urban congestion, and topological features of the islands that cause predictable and unpredictable delays. Ocean rescue times depend on winds, weather conditions, storm surges, and tides, as well as on the standby position of the sea ambulance at a distant island (Lipayran). Therefore, they are not proportional to nautical distance.

### Ambulance rescue time contours

DRRMO staff documented the shortest and longest rescue time, defined as the time required to load a patient at the scene onto an ambulance, transfer the patient to the District Hospital in Bantayan Town, and unload the patient at the emergency room. They also identified rescue times for outlying islets in the archipelago and included the time needed for port embarkation, sea ambulance transfer, port disembarkation, and land ambulance transfer.

The chiefs indicated that ambulance speeds depend on urgency, patient welfare, and road traffic. The slowest ambulance speed was 40 km/hr, while the fastest speed was 60 km/hr, on average. Maximum rescue times included allowance for unexpected but common delays, as expressed by ambulance drivers in the Bantayan DRRMO. We extended the process to outlying islands and islets, where the durations of minimum and maximum rescue times by sea ambulance were much longer than those by ground ambulance transfer on Bantayan Island.

A sea ambulance stationed at Lipayran Island 16 km from the Bantayan port responds to local island/islet emergencies. Transit from Lipayran Island to Bantayan Port requires 18 min with an open water net speed of 53.3 km/h. Distress calls from islets far from mainland Bantayan (e.g., Botigues, Doong, Mambacayao, Luyongbaybay, and Hilotongan Islands) require an additional 5–10 min. During rainy and windy days with ocean swells and other delays, island residents use sandboats/pumpboats, generating worst-case scenarios with an additional 30–45 min delay.

We highlighted the main ambulance routes on the nautical map of the Bantayan Archipelago and identified the primary EMS pathways. Next, we marked points where rescue times were equivalent and then interconnected them with splines of equivalent time intervals joined to form smooth temporal contours.

For Cebu Island, the Chief of the DRRMO in Bogo City provided details for ground ambulance rescue time. The average ambulance speed reached 100 km/hr with an additional 30-min delay due to traffic and road conditions when passing through urban regions. A 15-min delay was added to sites within and adjacent to Cebu City. The average of 100 km/hr plus a 30-min delay for congestion was used as the basis for constructing temporal contours showing ambulance rescue times from the northern and southern regions of Cebu Island to Vicente Sotto Memorial Medical Center, a level three Referral Hospital and Heart Center in Cebu City.

We used OpenStreetMap and Google Maps to validate Cebu Island distances. However, as in the Bantayan Archipelago, temporal contours were created from raw data obtained from expert local DRRMO staff. Summed travel increments generated minimum and maximum rescue times for archipelago connections to Cebu City.

### Population demographics

Coauthor S.T. and her public health team on Bantayan Island projected current demographics for the barangays and islands/islets of the Bantayan Archipelago and updated the most recent Philippines census of 2020. Population projections are posted online [38]. We used these data to identify the numbers of people living on Bantayan Island, surrounding islands, and small islets farther out in the archipelago.

### Statistics

Student’s t-test for mean paired differences was used to compare the fastest and slowest sea/land ambulance rescue times from identical points of origin to the Bantayan District Hospital. Island/islet and mainland barangay mean paired differences were analyzed separately. The Wilcoxon signed-rank test was used when the distribution of paired differences was not normal. The Shapiro–Wilk test was used to check normality of the paired differences.

## Results

### Geospatial orientation

**Fig. 1:**
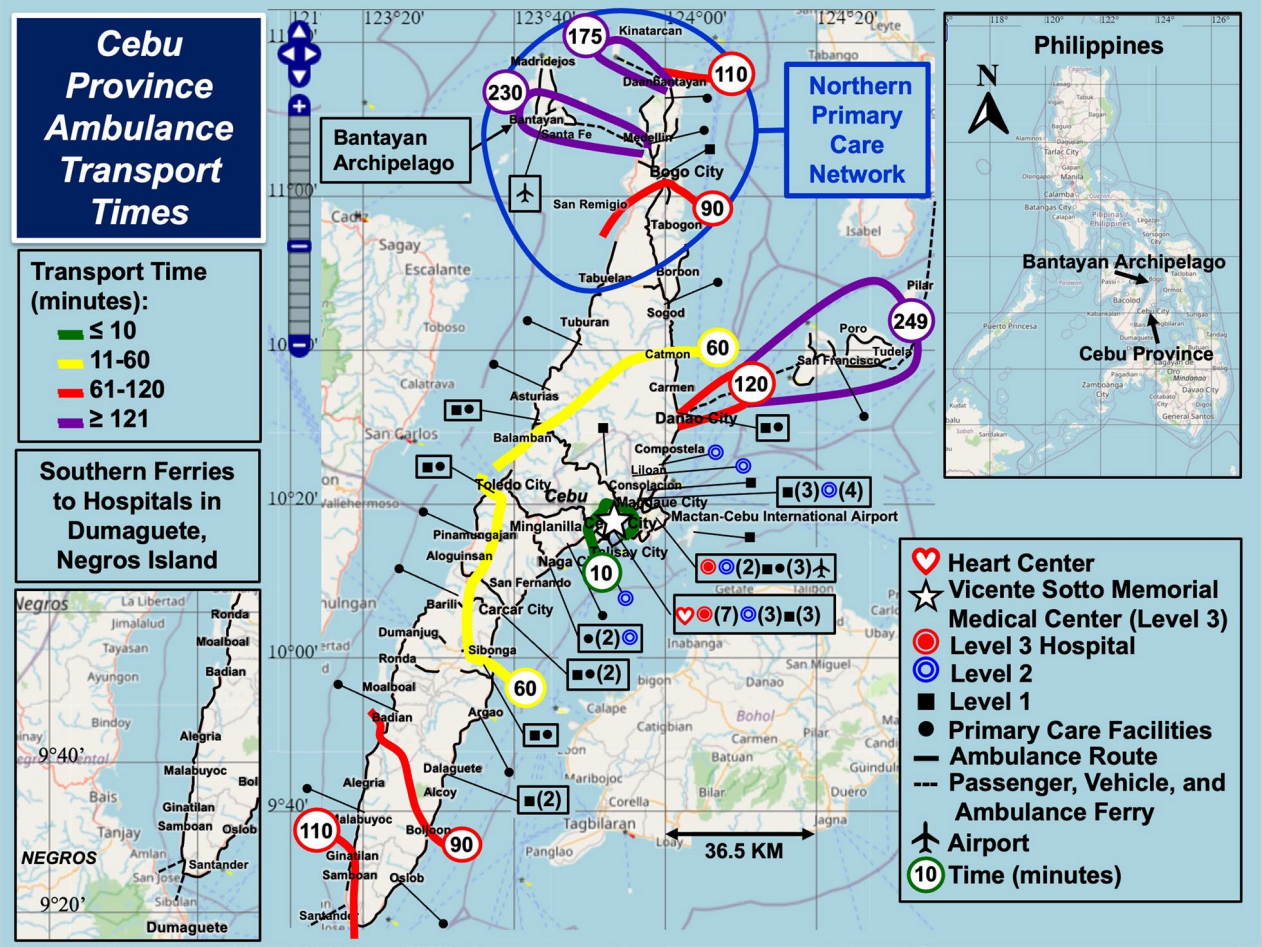
Cebu Province Ambulance Transport Times. The contours show ambulance rescue times to Vicente Sotto Memorial Medical Center in Cebu City. The Bantayan Archipelago is situated within the Northern Primary Care Network to the north of Cebu City

Figure 1 shows Cebu Province in the Visayas island group of the Philippines and the location of the Bantayan Archipelago to the northwest. Rapid rescue presents a fundamental problem for the archipelago, surrounding islands, and islets. In the southern tip of mainland Cebu, people often take a “shortcut,” that is, a ferry to Negros, the island to the west, to expeditiously seek critical care in Dumaguete.

Interventional cardiac care is limited to a few hospitals in Cebu City approximately 140 km southeast of Bantayan Town. These sites are outside the Northern Primary Care Network (see Fig. 1). The ferry from Bantayan Island to mainland Cebu is slow (90 min). Helicopter and fixed wing airplane transport are not generally available to the public but may be for the military or privileged few wealthy enough to pay high fares.

Narrow, windy, flash flood, and landslide-prone mountainous roads prolong rescue times on mainland Cebu. Challenges arise for emergency rescue and ambulance transfer from the Bantayan Archipelago to tertiary care in Cebu City (Table 1). Rapid care and diagnostic monitoring en route of critical conditions, such as acute myocardial infarction, may not be possible without restructuring public health approaches.

**Table 1:** Ambulance Rescue in the Bantayan Archipelago

Region or Site	Carriage	Service	Type	Vehicles/Vessels	Geospatial Coverage	POCT Instruments
1. Municipality^a^						
Madridejos	Rescue vehicle	On-scene rescue, transport, and basic life support	NC	3	Northern Bantayan Island	Pulse Oximeter
Bantayan	Same as above	Same as above	NC	7^b^	Central	Pulse Oximeter
Santa Fe	Same as above	Same as above	NC	2	Southern	Pulse Oximeter
2. District Hospital						
Referral hospital for the entire archipelago	Ambulance	Patient transport, basic or advanced life support depending on staff availability	I, II planned	2	Bantayan Island	Pulse oximeter, Glucose meter
3. Outer Islands						
Bantayan Port	Sea ambulance^c^ (outboard motor)	Basic life support, patient transport	NC	1 + 1 more planned^d^	Primarily southwest islands in the archipelago	Pulse Oximeter
Both ports	Sand Boat (very slow)	Back-up for rough weather	NC	(used as needed)	Both regions	None
Santa Fe Port	Sea ambulance (outboard motor)	Basic life support, patient transport	NC	1	Sea areas east of Bantayan Island (rarely private islands)	Pulse Oximeter
4. Potential Future Resources						
Helipads located at or near the District Hospital and on Doong Island	Helicopter – Air ambulance	From outer islands to District Hospital and from the archipelago to Cebu City	–	1	Sites of crises, emergencies, disasters, and epidemic outbreaks	Potential for onboard Dx, Rx, and live telehealth guidance
Air ambulance at the new airport located near Santa Fe	Fixed wing aircraft &/or sea plane air ambulances	Point-to-point critical in-flight support and fastest means of patient transport	–	1	From Bantayan Island to Cebu City or Manila based on diagnosis and therapeutic needs	Potential for diagnostics-guided therapy in flight

a) Each municipality operates a DRRMO that provides emergency assistance for the community. b) The Bantayan local government unit operates two vehicles for patient transport (e.g., by appointment) and two for transport to cities on mainland Cebu (Bogo, Danao, or Cebu City); three vehicles operated by the DRRMO respond to on-scene emergency calls but also provide patient transport within the municipality. c) The sea ambulance operates under the jurisdiction of DRRMO services. d) An additional sea ambulance will be acquired for navigating low tides. In low tides, the port of call changes to a sandbar southwesterly from Bantayan Island where a new dock will be built on the southern shoreline of Barangay Sulangan

*DOH* Department of Health, *DRRMO* Disaster Risk Reduction and Management Office, *Dx* diagnosis, *NA* not available, *NC* not yet classified by the DOH as either type I (basic life support) or type II (advanced life support), *POCT* point-of-care testing and *Rx* treatment

### Diagnostic testing sites

**Fig. 2:**
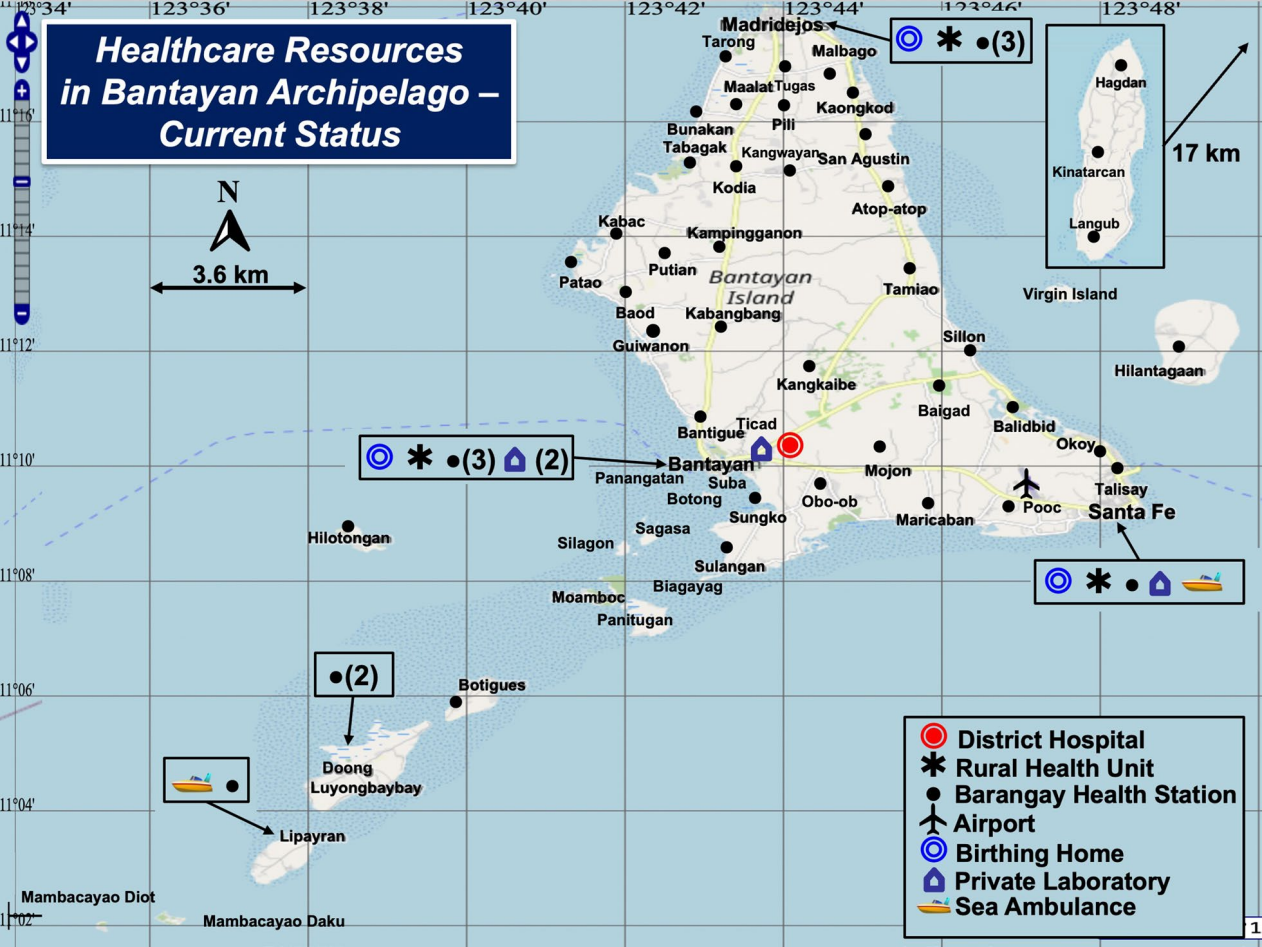
Healthcare Resources in the Bantayan Archipelago. This figure shows the distribution of healthcare resources in the small-world network of the Bantayan Archipelago. The District Hospital, a basic resource, is the main referral site on Bantayan Island

Table 2 documents diagnostic tests and instruments available at healthcare facilities in the Bantayan Archipelago. Figure 2 shows healthcare facility locations. Most diagnostic testing is basic, except where Rural Health Units use the GeneXpert for the molecular detection of tuberculosis, a grassroots program promoted by the government of the Philippines to mitigate local contagion (see “Illustrations of spatial care paths” section below). Barangay Health Stations provide primary care but in approximately half, diagnostics such as glucose meters lack test strips and cannot be used.

**Table 2:** Diagnostic Testing Performed at Geographic Sites in the Bantayan Archipelago

Geographic Site (N), Goal, and Hours	Diagnostic Tests			
	Hematology	Chemistry	Microbiology	Notes and Exceptions
Barangay Health Stations (49). Preventative care. (No beds.) M-F 8–5 and on call Saturday/Sunday	None	Glucose meter (operable when test strips are available), O_2_ saturation (pulse oximeter)	None	•27 (55.1%) Barangay Health Stations have glucose test strips •22 (44.9%) without strips cannot use meters
Birthing Homes (3). Midwives assist deliveries. Open 24/7	None	Pulse oximeter, glucose meter, pregnancy test	None	•Prenatal screening is performed in Rural Health Units
Rural Health Units (3). Preventative and primary care. M-F 8–5 and on call Saturday and Sunday	CBC, Hct, platelet count, blood typing (A, B, O, Rh)	O_2_ saturation (pulse oximetry), FBS/RBS, SGOT, SGPT, uric acid, Chol, Cr, BUN, UA, lipid panel, pregnancy test (urine beta-HCG)	COVID-19 RAgT, HIV, TB (GeneXpert), Dengue (NS1), stool exam (gross, bacteria, parasites, ova), syphilis, HBsAg	•Prenatal screening comprises CBC, FBS, HBsAg, and UA •Lipid panel — total cholesterol, triglycerides, HDL, and LDL •No blood gas testing • UTZ — availability depends on radiologists
District Hospital (1). ER, labor and delivery, limited surgery (e.g., C-section); local referral hospital with limited specialty services. 25 beds with reserve capacity to 55. M-Sat 8–5, on call evenings, Sunday	CBC, Hct, platelet count, HbA1c, blood typing	Electrolytes^a^ (Ca^++^, K + , Na^+^, Cl^−^), Glu, FBS, OGTT, ALT, AST, uric acid, Chol, lipid panel, Cr, BUN, UA, pregnancy test. Glucose meters and pulse oximeters in the ER	COVID-19 RAgT, RT-PCR. HIV, *Dengue* (duo rapid test), *Salmonella typhi* (rapid test), stool exam, FOB, syphilis, HBsAg, HCV screening test	•Qualitative cTnI testing^b^ is performed in lab only when reagents are available. [No cTnT.] •Blood gas testing (pO_2_, pCO_2_, pH) not available • Genrui^c^ instrument for cTnI, NT-proBNP, CK-MB, and myoglobin testing not used – no reagents
Private Laboratory				
Oasis. Referral lab and limited diagnostic care. M-Sat 7–6. Closed Sundays	CBC and blood typing on site	Pregnancy test (serum) and UA on site	Syphilis and stool exam on site	•UTZ for pregnancy •Branch lab outsources other tests to main lab in Bogo, Cebu Province

a) Genrui (Shenzhen, China). Electrolyte Analyzer GE 300. Ion-selective instrument that measures ionized calcium (Ca + +), potassium, sodium, and chloride. https://www.genrui-bio.com/products/electrolyte-analyzer-ge300.html Accessed November 22, 2023

b) InTec (Fujian, China). Qualitative One-step (15–20 min) Troponin I Test Card (sensitivity not specified; whole blood, plasma, or serum). https://www.intecasi.com/rapid-troponin-i-test_p67.html. Accessed November 22, 2023

c) Genrui (Shenzhen, China). Quantitative Immunoassay Analyzer FA50. https://www.genrui-bio.com/products/quantitative-immunoassay-analyzer-fa50.html Accessed November 22, 2023

24/7, service all days/hours; *ALT* alanine transaminase (SGPT, serum glutamic pyruvic transaminase), *AST* aspartate transaminase (SGOT, serum glutamic oxaloacetic transaminase), *BUN* blood urea nitrogen, *CBC* complete blood count, *Chol* cholesterol, *CK-MB* creatine kinase MB isoenzyme, *COVID-19* Coronavirus infectious disease 2019, *Cr* creatinine, *cTnI/T* cardiac troponin I/T, *ER* emergency room, *FBS* fasting blood sugar, *FOB* fecal occult blood, *HBsAg* hepatitis B surface antigen, *HbA1c* hemoglobin A1C, *HCG* human chorionic gonadotropin hormone, *Hct* hematocrit, *HDL* high density lipoprotein, *HIV* human immunodeficiency virus, *LDL* low density lipoprotein, *Myo* myoglobin, *NS1* Non-Structural Protein 1, NT-proBNP, *NT-proB* type brain natriuretic peptide, *OGTT* oral glucose tolerance test; *RAgT* rapid antigen test, RBS, random blood sugar, *RT-PCR* reverse transcription polymerase chain reaction, *TB* Tuberculosis, *UA* urine analysis, and *UTZ* ultrasound

Testing on board ambulances is generally limited to pulse oximeters that measure oxygen saturation continuously and glucose meters that measure discrete capillary glucose levels, but test strips may not be available. Sea ambulances have an ambulance stretcher, automated external defibrillator (AED), suction machine, nebulizer, aneroid sphygmomanometer, stethoscope, oxygen cylinder, and examining light. Testing for COVID-19 must wait until patients arrive at larger healthcare sites, such as Rural Health Units that perform rapid antigen tests (RAgTs) and the District Hospital, which offers RAgTs and RT-PCR assays, the latter capable of both ruling in and ruling out SARS-CoV-2 infection as prevalence increases [8].

The District Hospital in Bantayan has an active emergency room that offers the laboratory test menu in Table 2. Specimens referred there may sit idle because reagents are not available. Important tests, such as quantitative cardiac troponin I and T, brain natriuretic peptide (BNP, NT-proBNP), and other cardiac biomarkers cannot be performed. There is no whole-blood analyzer for measuring blood gases and pH on Bantayan Island. Satellite private laboratories perform limited chemistry tests and refer specimens to Bogo City laboratories to the east on mainland Cebu with turnaround times of up to days.

### Rescue time contours

**Fig. 3:**
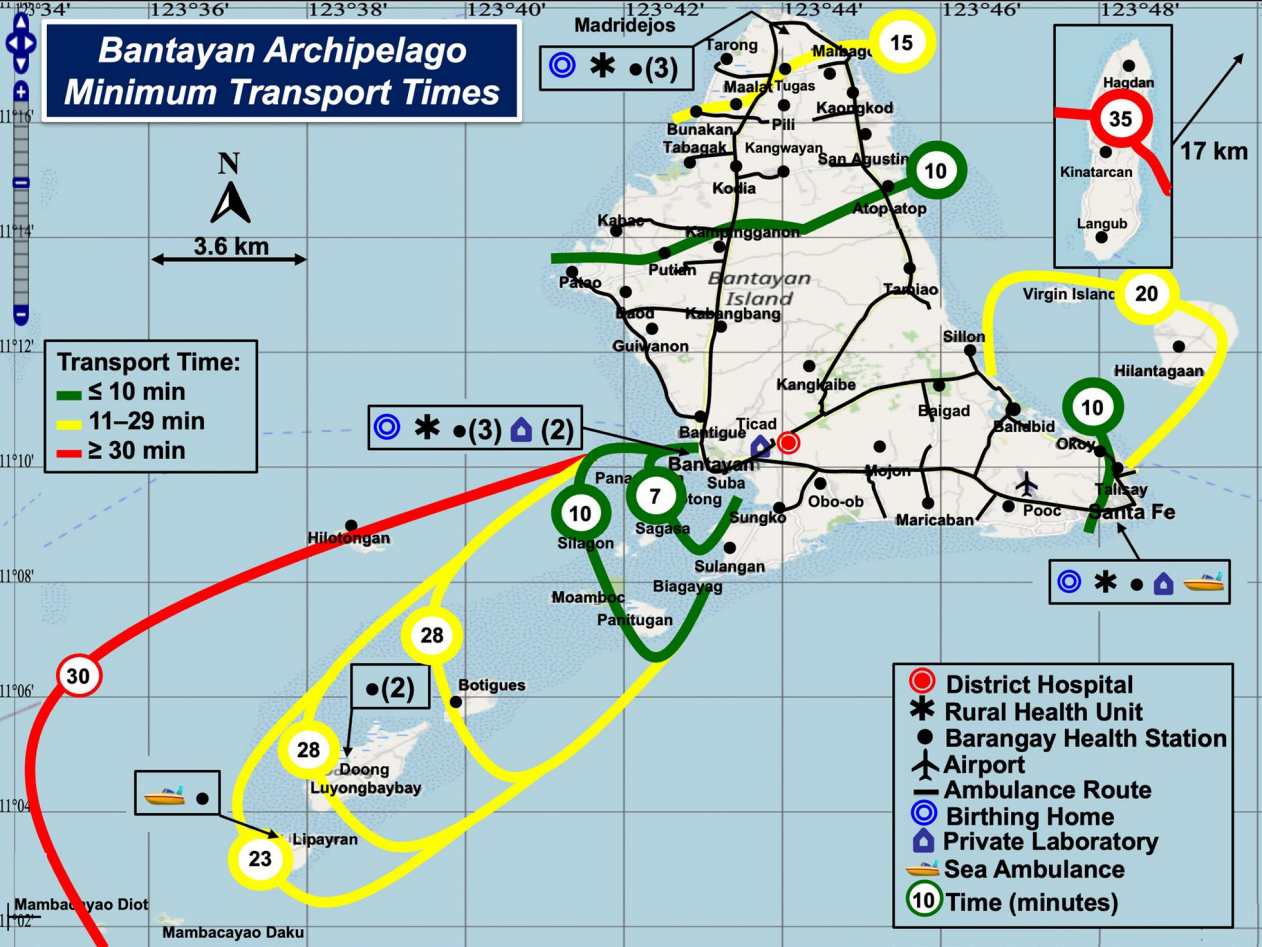
Bantayan Archipelago Minimum Transport Times. Rescue times become progressively longer radiating out from the District Hospital. Time intervals were determined from raw data provided by sea and land ambulance operators

**Fig. 4:**
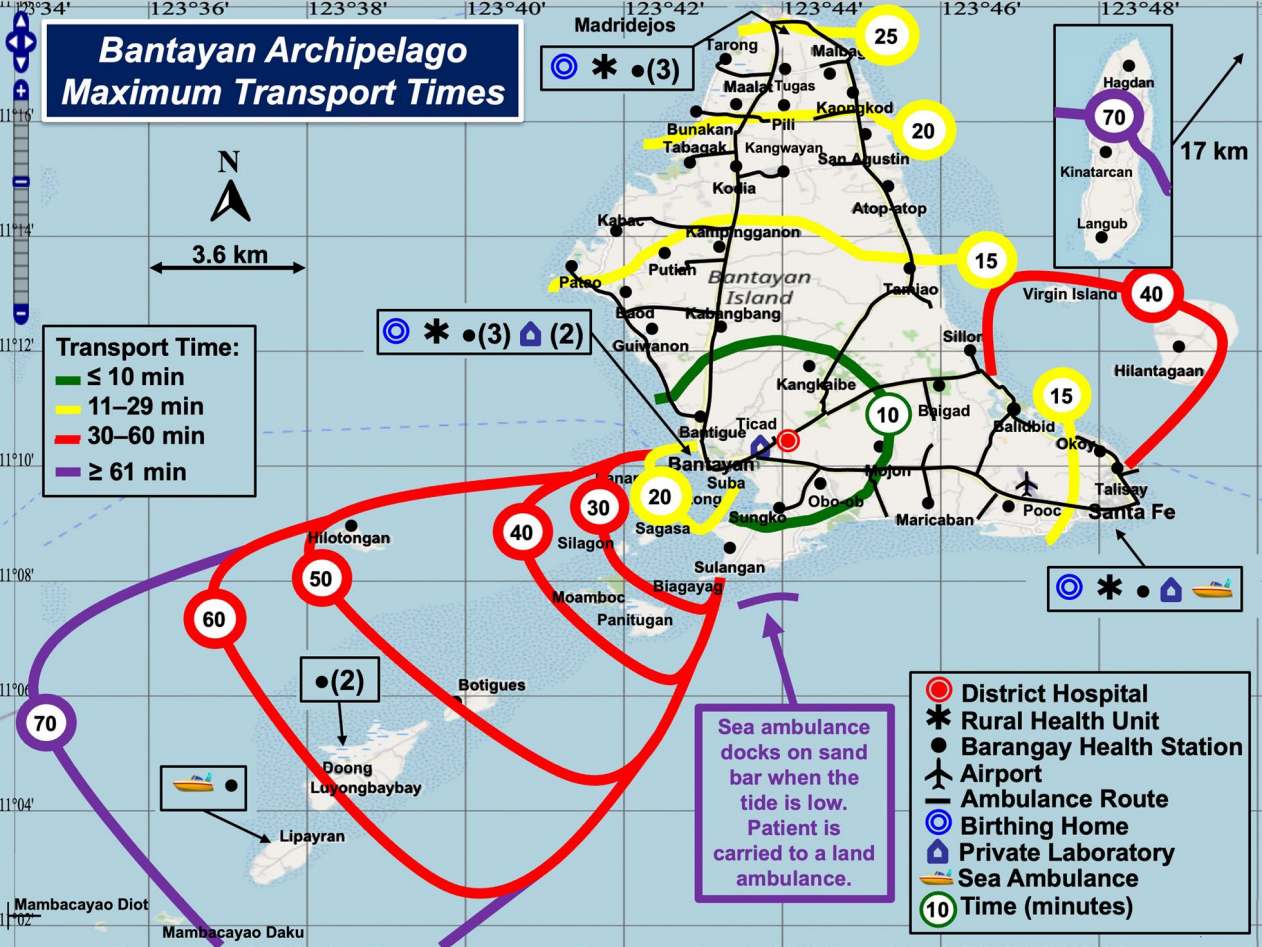
Bantayan Archipelago Maximum Transport Times. During low tides, the port of call changes to a sandbar southwest from Bantayan Island. Patients must be hand-ported from the sea ambulance to the land ambulance waiting onshore

Rescue services are well organized (see Table 1). However, natural geography, topography, and inclement weather prolong sea and land ambulance transfers. Figures 3 and 4 show minimum and maximum patient rescue times with color-coded contours. During bad weather, tides interfere with sea ambulance operations. Rescue personnel use an alternate docking site toward the southwest of Bantayan Island (see Fig. 4). Personnel must then carry the patient to a ground ambulance for safe rescue.

The median (range) and mean (SD) fastest sea/land ambulance rescue times (minutes) from islands and islets (N = 19) to the District Hospital on mainland Bantayan Island are 20 (7–35) and 18.4 (10.1) and the slowest, 40 (17–75) and 44.3 (18.2), respectively. The mean paired (slow-fast) difference in island/islet rescue times (N = 19), is 25.8 (10.3) minutes (P < 0.0001) using Student’s t-test for paired differences (t = 10.9, no outliers observed). The differences were distributed normally.

The median (range) and mean (SD) fastest land ambulance rescue times (minutes) from Bantayan mainland barangays (N = 40) to the District Hospital were 10.6 (5.7–17.4) and 11.0 (2.78) and the slowest, 14.5 (8–26.1) and 16.0 (4.30), respectively. The distribution of paired differences in slowest minus fastest rescue times was bimodal (nonnormal) because 19 barangays are near the District Hospital or on the main north–south road, and paired differences in rescue times for those sites were < 3 min. Nonparametric analysis of all 40 paired differences in barangay rescue times using the Wilcoxon signed-rank test showed P < 0.0001.

### Illustrations of spatial care paths

#### Acute myocardial infarction

**Fig. 5:**
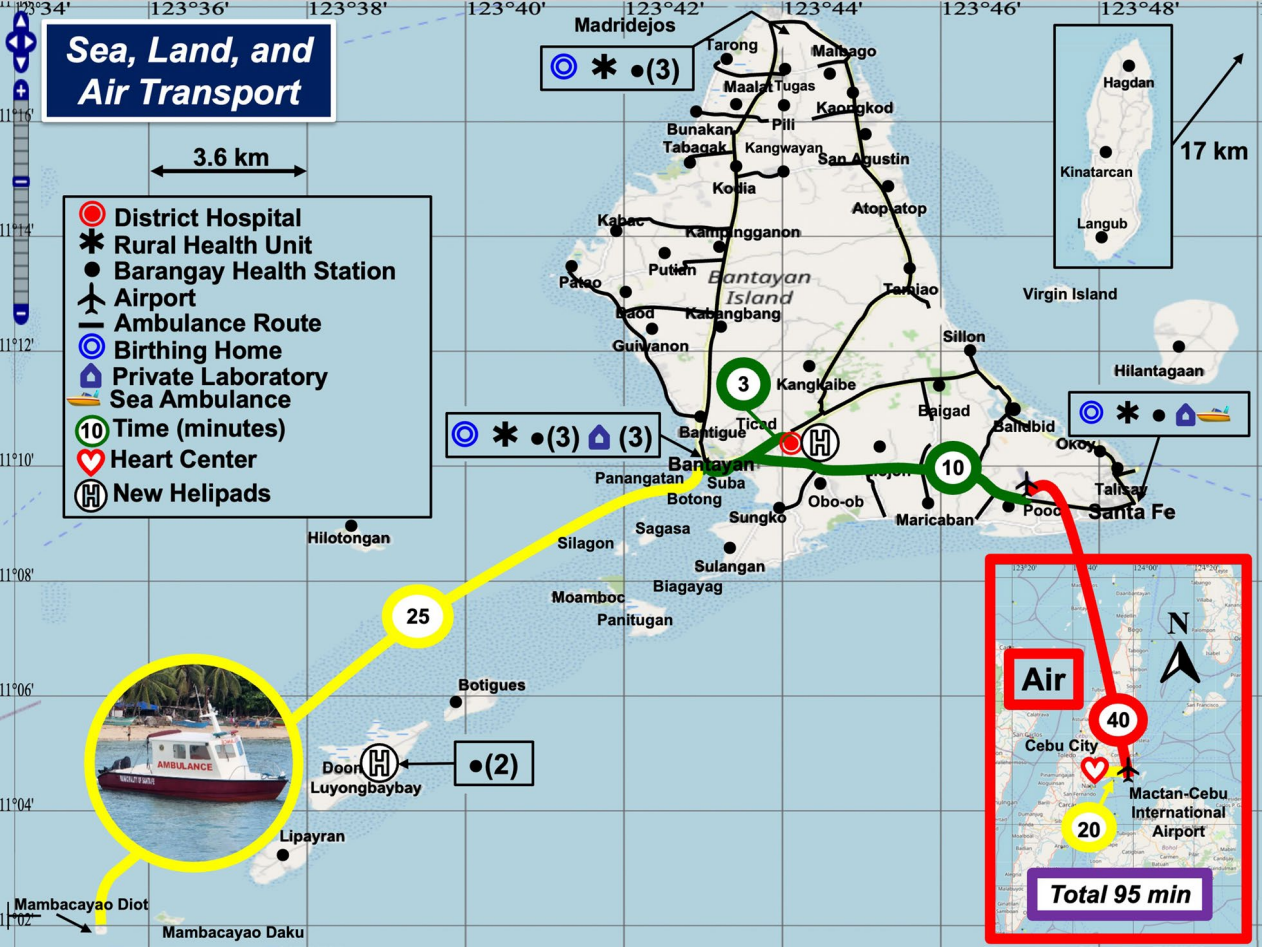
Spatial Care Paths for Acute Myocardial Infarction. A patient with acute myocardial infarction must endure a total transit time of 95 min (purple). If transported directly to the District Hospital via the spline (green) from the port (3 min), the total transit time would be < 30 min

Spatial care path analysis for patients who reside on the southwest islands and islets of the Bantayan Archipelago and have acute chest pain revealed a serious problem (Fig. 5). There are four time-consuming travel legs: a) sea ambulance from the islet to the port in Bantayan Town (yellow), b) land ambulance from the port to the airport (green), c) fixed wing air transport to the Cebu-Mactan International Airport (red), and d) land ambulance to Vicente Sotto Memorial Medical Center in Cebu City (yellow).

Suppose an elevated cardiac troponin were documented by sea ambulance operators (if equipped to perform the test) and their crew. Then, the minimum total rescue time of 95 min to Vicente Sotto Memorial Medical Center, a Level 3 hospital in Cebu City, is excessive even if a cardiologist monitors the ECG remotely so that the ambulance can bypass local health facilities en route to fixed wing aircraft transport to Cebu City.

For an expeditious transfer of 28 min (25 min by sea, 3 min on land), which might be lifesaving, the spatial care path should be directed to the Bantayan District Hospital via the short green 3-min connector from the port in Fig. 5. Elevated cardiac troponin on the sea ambulance would alert the catheterization team to prepare, further improving efficiency, increasing chances of survival, and elevating the local standard of care. However, interventional care by a cardiologist would require a significant upgrade of specialists and supporting personnel, facilities, and equipment at the Bantayan District Hospital.

**Fig. 6:**
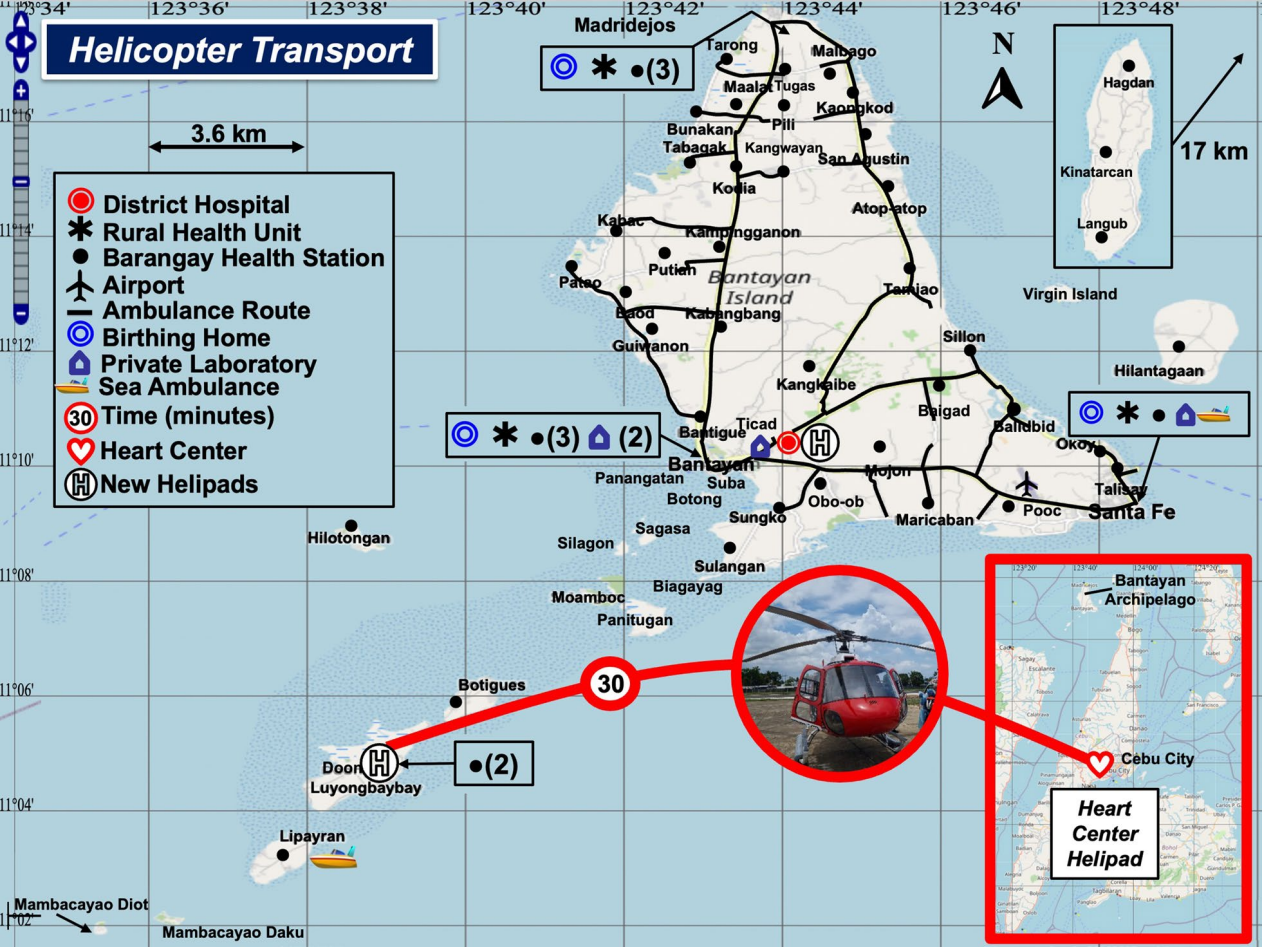
Helicopter Transport. Helicopters and helipads are not currently available in the Bantayan Archipelago. However, direct helicopter transport could decrease the rescue time from islands/islets to Heart Centers in Cebu City to 35 min. This figure shows two recommended sites for island helipads

Another option would be to transport the patient directly to a heart center in Cebu City by helicopter (Fig. 6). A nurse could perform critical care testing onboard the helicopter. Unfortunately, helicopter service is not available, and inclement weather conditions would ground the helicopter as well as fixed wing aircraft (see Fig. 5). Hence, the most reliable spatial care path calls for an upgrade of the District Hospital with interventional care by a cardiologist.

#### Hypo- and hyperglycemia, HbA1c, and therapeutic monitoring

Sea and land ambulances in the Bantayan Archipelago are equipped with glucose meters. Although most glucose meters are not capable of producing accurate quantitative results when glucose is very low or high [39–41], ambulance operators may discover acute hypo- or hyperglycemia during the first encounter with the patient and quickly establish a spatial care path to the nearest emergency facility where laboratory testing (if available) can quantitate the glucose abnormality. If comatose, the patient will require rapid evaluation and possibly administration of 50 mL of 50% dextrose.

We could not find HbA1c testing in Barangay Health Stations or Rural Health Units for diabetes screening and therapeutic monitoring at the time of the needs assessments. However, the Municipality of Bantayan purchased an instrument for HbA1c and started measurements at a clinic site in December 2022. Mobile HbA1c testing on a van that rotates to sites near patient homes, Barangay Health Stations, and workplaces would move decision making up the spatial care path nearer homes and patients, which would avoid downstream delays transporting specimens and receiving results, thus saving time and expense away from productive work to travel to distant testing sites.

#### COVID-19 and other infectious diseases

Community access to COVID-19 testing was limited with no self-testing or saliva testing programs in place. RAgTs were not performed on ambulances but are needed to help protect rescue personnel and warn destination emergency room staff. Rarely, people can obtain RAgT kits for testing at home, but there is no government distribution program. Barangay Health Stations lacked COVID-19 testing. Rural health units offered COVID-19 RAgTs. The District Hospital laboratory performed RT-PCR testing. Travel time to the District Hospital from outlying islands (see Fig. 4) for confirmation of RAgT results is quite prolonged.

Spatial care paths start at discrete sites where people first find diagnostic portals [8]. With a pattern of limited COVID-19 testing, delays in submitting swab and other types of specimens and receiving results can spread contagion. Testing could be moved out into the community. Even children are capable of reliable self-testing [42, 43].

Mobile testing on vans rotating throughout the community would improve access. Spatial flexibility ensures access for specimen procurement and possibly direct POCT for other infectious diseases, such as influenza A/B, HIV, dengue, and monkeypox. Test kit vending machines could be placed near municipal halls in the community. We illustrate these exciting point-of-need modalities below.

#### Tuberculosis peripheral POCT (ongoing)

The United States Agency for International Development and the Bantayan Public Health team led by coauthor S.T. launched peripheral POCT for tuberculosis to benefit at-risk, geographically isolated, and disadvantaged islanders, part of the “introducing New Tools Project” being rolled out in nine high burden countries, including Cambodia, Vietnam, and the Philippines in Southeast Asia [44].

POC technologies comprise: a) a battery-operated, lightweight, low radiation exposure Fuji X Air Ultraportable Chest X-ray (UP CXR) with artificial intelligence-aided read-out; b) the TrueNAT (Molbio, Gao, India), a portable battery-operated instrument performing molecular detection of *Mycobacterium tuberculosis* and rifampicin resistance in one hour; and c) patient self-video monitoring to assure medication compliance [45, 46].

Molecular detection is intended for the peripheral level, that is, community health facilities and mobile clinics. It uses the first WHO-recommended rapid molecular test for detection of tuberculosis and rifampicin resistance. In the Bantayan Archipelago, the percent of confirmed cases was 8.9% (158/1,774) with a range of 1.3 to 17.6% in 84% (21/25) of barangays where testing was performed in the Phase I 2022 study [46]. Characteristic of a spatial care path, the people residing in outlying islands and islets access diagnostics and therapeutics locally, rather than traveling to Bantayan Island.

## Discussion

### Geospatial solutions for climate change crises

**Fig. 7:**
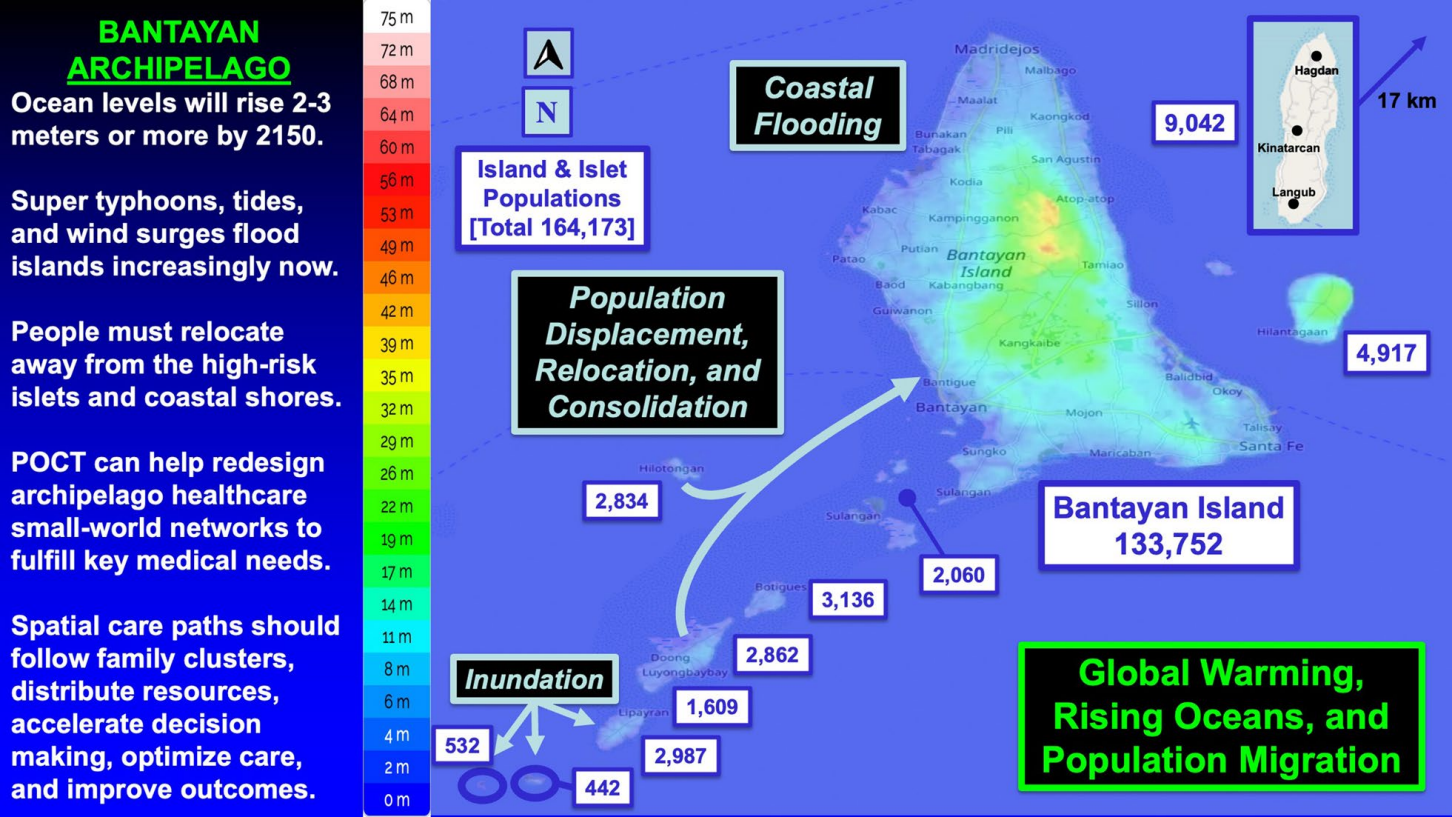
Global Warming, Rising Oceans, and Population Migration. Climate change, rising oceans, and population migration demand changes in the way emergency healthcare services are delivered

To our knowledge, this research is the first to perform geospatial analysis of an island archipelago, design rapid response strategies for it, and address public health resilience in preparation for climate change crises. It is also the first to introduce geographic contour mapping for diagnostics planning in the Philippines, an island nation where healthcare access remains problematic for people distant from tertiary care. Rising oceans threaten not only the Bantayan Archipelago (Fig. 7) but also other islands in the Philippines.

Island nations are at risk. The Maldives have a maximum height of only 2.4 m (7 feet, 10 inches), and Indonesia is inventorying its 17,508 islands (6,000 inhabited) because some are disappearing [47]. Progressively stronger and more frequent super typhoons portend increased acute isolation and give real urgency to the deployment of POC strategies that create local and regional geographic autonomy for crisis responses and recovery operations. These same strategies have utility to improve the accessibility of daily urgent care for island families.

### Benefits of point-of-care strategies

Ferguson et al. [48–50] and Kost [51] showed that geospatial analyses derive high impact by improving alternative diagnostic placement strategies in limited-resource settings and by revealing deficiencies in healthcare access pathways. In limited-resource Isaan and other Thai provinces, Kost et al. [29] innovated early upstream POC cardiac troponin testing on spatial care paths that expedited transfers directly to hospitals capable of intervening and improved outcomes following coronary occlusion. These spatial efficiencies were also recommended for Central Vietnam [16].

Studying Vietnamese healthcare small-world networks, Ventura et al. [34] concluded that public health should fund enhanced access to POC diagnostics for diabetes. Nguyen et al. [35] recommended that POC coordinators manage bedside testing, quality assurance, and independent accreditation. During visits in the Visayas we encountered only one POC coordinator at Vincente Sotto Memorial Medical Center, a referral hospital in Cebu City. None were employed in the Bantayan Archipelago, a deficiency that should be corrected.

In sparsely populated Australia, Hengel et al. [53] found that, “…decentralized POCT should be considered for communities in need, especially those that are undertested and socially vulnerable” and that “…decentralized testing should be part of the core global response towards suppressing COVID-19.” In Ghana, Kuupiel et al. [53, 54] recommended geographic accessibility of POC diagnostics for tuberculosis, glucose-6-phosphate dehydrogenase deficiency in antenatal care, and hypertensive disorders of pregnancy.

Jafri et al. [56] devised a framework for comprehensive, integrated, and networked POCT with professional supervision in low-resource countries. For clinical services in New Zealand where the guiding principles are to improve health access and medical outcomes for the island nation, Herd and Musaad [57] concluded that POCT in rural and remote settings can be rewarding for clinicians and an “…important component of the health system to improve outcomes and reduce inequity…now and in the future.”

### Role of prehospital testing

Investigators emphasized the need for prehospital testing in island communities. Fernando et al. [58] evaluated a rapid diagnostic test for the detection of malaria when preventing reestablishment in Sri Lanka. Oviedo et al. [59] combined serological, antigen detection, and DNA data for *Plasmodium falciparum* to generate robust geospatial estimates of malaria transmission in Haiti with a confident description of its spatial epidemiology. In the same setting, Rogier et al. [60] showed that rapid diagnostic tests for *Plasmodium falciparum* in community settings had a sensitivity of 86.3–96.0% and a specificity of 90.0–99.6%.

In 2022 no cases of malaria were documented in the Bantayan Archipelago, but eighty-nine cases of dengue fever appeared, which underscores the need for mobile dengue testing (e.g., NS1, IgG, IgM) to minimize epidemiological dissemination and warn of potential hemorrhagic fever that may require hospitalization, hourly bedside hematocrit monitoring, and intensive transfusion support. Since the last major compilation of POC technologies for disasters, emergencies, and public health crises in 2015 [61], prehospital diagnostics have blossomed and increased the efficiency and effectiveness of healthcare systems.

Prehospital diagnosis improves outcomes in numerous medical conditions. Please see the righthand column of the Compendium, Additional file 1. Prehospital diagnosis can help optimize healthcare by mobilizing diagnostic solutions where shared resources must be conserved. The benefits will accrue not only from more efficient urgent and emergency care but also from an improved safety net for tourism and other activities that promote the local economy and help justify the costs of implementation.

### Environmental controls, training, and competency

Appropriate environmental controls, personnel training, and professional competency will help realize the full benefits of POCT in limited-resource settings. Manufacturers standardly issue requirements for operational and storage temperature and humidity. Exceeding these limits, even for brief periods of time, can render devices inoperable or produce clinically significant changes in POC test results [30, 62–76]. This year, the International Federation of Clinical Chemistry Committee on POCT published guidelines for testing outside the hospital [77]. The guidelines emphasize managing risk proactively to ensure reliable results.

Staff training and competency enhance POCT performance [78–81]. Stavelin and Sandberg [82] recently addressed analytical performance specifications for POCT and concluded that in primary healthcare they can differ from those in the clinical laboratory because of intended uses, response times, and geographic factors, but emphasized the need for wise instrument selection, user training, and quality assurance for optimal care. Public health organizations implementing POCT strategies should carefully review and follow these guidelines and recommendations to ensure that the accuracy of POCT is not compromised [77, 82, 83].

## Conclusions and recommendations

Table 3 summarizes our recommendations for the Bantayan Archipelago. The recommendations are supported by needs identified during field visits, professional input from local public health officials, geographic contour map patterns, the spatial care path designs for the Bantayan Archipelago, and outcomes evidence in the Compendium (Additional file 1). Additionally, successful mobile van testing (Table 4) during COVID-19 in Vacaville, California, the site of the first community transmission in the United States, demonstrated that a mobile laboratory can deliver badly needed urgent and wellness services. The mobile laboratory also allows control of an appropriate environment for instrument operation and reagent storage.

**Table 3:** Recommended Patient-focused Diagnostics for Spatial Care Path Acceleration in the Bantayan Archipelago

Medical challenge	Diagnostics	Implementation sites for rapid decision making	Enhanced outcomes
Acute abdomen	CBC, enzymes (ALT, AST, lipase), ECG, abdominal UTZ	Rural Health Unit triage diagnostics with progressive plan for District Hospital support of transfer to tertiary care medical facility	Diagnose and stabilize patients with acute abdomen (e.g., appendicitis, pancreatitis,) before transferring to a tertiary medical facility
Acute myocardial infarction	POC qualitative or semiquantitative cTnI/ cTnT	Ambulance, Barangay Health Station, and Rural Health Unit rule in of AMI	Fast therapeutic turnaround time, earlier triage, bypass time-consuming intermediate processing steps, appropriate and timely therapy (e.g., CABG, PCI), improve survival, and diminish mortality in rural versus urban environments
	Quantitative cTn, hs-cTnI or hs-cTnT	Rural Health Unit, District Hospital, or heart center for both rule-in and rule-out of AMI with Rx in < 30–60 min	
COVID-19	POC RAgT, self-testing, home LAMP tests (free)	Ubiquitous access: vending machines, mobile vehicles (ambulance, health van), Barangay Health Stations, Rural Health Units, pharmacies, and more	Self-testing empowerment, early detection, family and workforce protection, outbreak mitigation, patient education, new public health paradigm, and precedents for the next pandemic
	RT-PCR	Rural Health Unit, District Hospital	
	Viral load	Academic or reference laboratory	
Critical care Support and transport	O_2_ saturation (pulse oximetry), electrolytes, BG, Ca^++^, and pH	Sea, land, and air ambulances	Pulmonary and cardiac support, decreased morbidity and mortality
		Rural Health Units, emergency rooms	
	Glucose, lactate PT (INR), D-dimer	Prehospital transport and ambulance services	Risk stratification, spotting critical patients, triage to intensive care, hemostasis Rx, PE management
Prediabetes, diabetes diagnosis, and therapeutic monitoring	HbA1c near homes every three months	Rotating POC HbA1c instruments in community sites during physician rapid Dx and Rx on site	Earlier Dx of prediabetes and diabetes, improved control of patients under Rx
	Critical care test clusters selected from the POC tests above	Ambulances for immediate detection of hypoglycemia and hyperglycemia	Risk mitigation using onsite Dx of diabetic ketoacidosis, hyper-glycemic hyperosmotic coma, and other glucose hemostasis problems
“Hidden” medical problems	POC ultrasound	Sea, land, and air ambulances, primary care network, ER/EDs, and Barangay Health Stations in the surrounding islets and islands	Numerous applications for triage, acute care, intubation, and orthopedics
Infectious diseases	POC STI (STD), Dengue, EVD, Monkeypox, and other rapid tests	All points of need depending on deadly outbreaks and community prevalence of sexually transmitted diseases (e.g., Monkeypox)	Rapid Dx, decreased opportunity costs, improved infant welfare, earlier start of isolation, and decreased time to Rx
Non-life-threatening acute conditions	O_2_ saturation, electrolytes, glucose, BUN, Cr, BNP (or NT-proBNP), portable ECG	Paramedic outreach to the patient home, community center, and Barangay Health Station	On site Dx, triage, and Rx of CHF and COPD exacerbations, dehydration, UTI, and other acute conditions in or near homes to spare EMS resources, avoid overload of ERs, and allow faster response for more pressing cases
Obstetric emergencies	CBC, glucose, ECG, pelvic UTZ, CTG	Coordinated strategy by Barangay Health Station, Rural Health Unit, and District Hospital to supply fetal heart monitoring along with basic POC tests already available on site	Decrease infant and maternal mortality resulting from obstetric complications (e.g., prolonged labor, placenta previa, and uterine atony)
Sepsis and septic shock	Test clusters drawn from suitable instruments (e.g., Cobas h232, HemoCue, i-Stat, StatStrip, CoaguChek, and Pocket-chem)	Land ambulance for rescue with on scene victim evaluation by means of a kit of POCT handheld instruments	Change in conveyance decisions, minimized rescue time, recognition of critical medical problems requiring admission and Rx, economic benefits, enhanced survival and lower morbidity and mortality
	Molecular diagnostics	District Hospital rapid molecular detection of pathogens (handheld and portable molecular diagnostics)	
Snake bite	PT (INR), aPTT, Hct/Hgb	Barangay Health Stations (depending on local prevalence of snake bites in the primary care network)	Rapid identification of coagulopathy, especially in children, and administration of antivenom
Stroke	CT scanner with POCT	Stroke ambulance	Rapid discovery of ischemic stroke, earlier administration of thrombolytics, transport directed to stroke centers without delay, and improved outcomes
Transfusion support	POC Hct/Hgb, blood typing (A, B, O, and Rh)	Drone-facilitated delivery of blood products to outlying islands, transport of specimens for diagnostic tests	Fundamental life support where it is lacking due to isolation in emergency situations
Tuberculosis	Fuji X Air ultraportable digital CXR with AI + battery-powered POC TrueNAT molecular Dx instrument	Mobile van and rotating peripheral healthcare screening for TB and rifampicin resistance in the community. USAID TB Initiative. First WHO-recommended rapid (1 h) molecular test for the detection of *Mycobacterium tuberculosis* complex bacteria and rifampicin resistance	Unique case finding program underway already finds ~ 9% infected with TB of first 432 patients screened in the Bantayan community. Low-cost testing with minimal biosafety requirements. Computer-aided detection of TB on CXR using artificial intelligence

*AI* artificial intelligence, *ALT* alanine transaminase (SGPT, serum glutamic-pyruvic transaminase), *AMI* acute myocardial infarction, *aPTT* activated partial thromboplastic time, *AST* aspartate aminotransferase (SGOT, serum glutamic oxaloacetic transaminase), *BG* blood gases (pO_2_, pCO_2_, pH), *BNP* blood natriuretic peptide (or NT-proBNP), *BUN* blood urea nitrogen, *Ca*^*++*^ ionized calcium [must be measured with a (portable) blood gas or whole-blood analyzer], *CBC* complete blood count, *CABG* coronary artery bypass graft, *CHF* congestive heart failure, *COPD* chronic obstructive pulmonary disease, *COVID-19* Coronavirus infectious disease 2019, *Cr* creatinine, *CTG* cardiotocography (continuous recording of the fetal heart rate obtained via an ultrasound transducer placed on the mother's abdomen), *cTn* cardiac troponin I (cTnI) or T (cTnT), *CXR* chest X-ray, *Dx* diagnosis, *ECG* electrocardiogram, *ED* emergency department, *EMS* emergency medical services, *ER* emergency room, *EVD* Ebola virus disease, *HbA1c* hemoglobin A1c, *Hct* hematocrit, *Hgb* hemoglobin, *hs-cTn* high sensitivity cTn, *INR* International Normalized Ratio, *LAMP* loop-mediated isothermal isolation, *NAAT*, nucleic acid amplification test, *PCI* percutaneous coronary intervention, *PE*, pulmonary embolism, *POC*, point-of-care, *POCT* POC testing, *PT* prothrombin time, *RAgT* rapid antigen test, *RT-PCR* reverse transcriptase-polymerase chain reaction, *Rx* treatment, *STI*, sexually transmitted infection (STD, ST disease); *TB* tuberculosis, *USAID* United States Agency for International Development, *UTZ*, ultrasound, *WBA* whole-blood analyzer, and *WHO* World Health Organization

**Table 4:** Rapid POC Tests Performed in Geographically Mobile and Pop-up Laboratories

Ranked by Analysis Time (min) FDA Status Hyperlink	Instrument, Manufacturer, Website Method, Test Type, Swab, Read	Specimen	Storage Temperature (°C) Testing Temperature (°C)	Sensitivity (%) Specificity (%)
**10** FDA EUA https://www.fda.gov/media/142916/download	*CareStart* COVID-19 Antigen Test Access Bio, Inc https://accessbiodiagnostics.net/carestart-covid-19-antigen/ Lateral Flow Assay, Antigen Test, Polyester Spun Nasal Swab, Visually Read	Anterior Nasal Swab/Nasopharyngeal Swab	1 ~ 30/15–30	87.18, 100 (AN); 93.75, 99.32 (NP)
** < 15** FDA CLEARED https://www.cdc.gov/flu/professionals/diagnosis/table-ridt.html\	McKesson Consult Influenza A & B Test McKesson Medical-Surgical Inc https://mms.mckesson.com/product/1076728/McKesson-Brand-181-36025 Antigen Test, Nylon Flocked Swab, Visually Read	Nasal Swab Nasopharyngeal Swab	2–30 /18–30	Flu A (NS): 91.7, 75.2 Flu B (NS): 82.4, 88.3; Flu A (NP): 89.6, 77 Flu B (NP): 86.8, 92.9
** < 15** FDA EUA https://www.fda.gov/media/139789/download	Assure COVID-19 IgG/IgM Rapid Test Assure Tech (Hangzhou Co., Ltd) http://www.diareagent.com Lateral Flow Chromatographic Immunoassay, Antibody Test, N/A, Visually Read	Whole Blood Serum, Plasma	2 ~ 30/15–30	88.7, 93.3
**15** FDA EUA https://www.fda.gov/media/137886/download	Quidel Sofia SARS Antigen Fluorescent Immunoassay Quidel Corporation https://www.quidel.com/immunoassays/rapid-sars-tests/sofia-sars-antigen-fia Immunofluorescence-based Lateral Flow Assay, Antigen Test, Foam Tipped Swab, Instrument Read	Nasal Swab	15–35	96.7, 100
**15** FDA EUA https://www.fda.gov/media/142701/download	Quidel Sofia 2 Flu + SARS Antigen Combo Fluorescent Immunoassay Quidel Corporation https://www.quidel.com/immunoassays/sofia-2-flu-sars-antigen-fia Immunofluorescence-based Lateral Flow Assay Antigen Test Foam Tipped Swab Instrument Read	Nasal Swab, Nasopharyngeal Swab	15–35	Flu A (NS): 90, 95 Flu A(NP): 97.1, 94.6 Flu B (NS): 89, 96 Flu B (NP)—90, 97 SARS-CoV-2: 95.2, 100
**20** FDA EUA https://www.fda.gov/media/151212/download	INDICAID COVID-19 Rapid Antigen Test PHASE Scientific International Ltd https://phasescientific.com Lateral Flow Immunoassay, Antigen Test, Nylon Flocked Swabs, Visually Read	Nasal and Nasopharyngeal Swab	2–30/15–30	84.4,96.3
** < 30** FDA EUA https://www.fda.gov/media/136345/download	Accula SARS-CoV-2 Test Mesa Biotech, Inc https://www.mesabiotech.com Reverse-Transcription Polymerase Chain Reaction & Lateral Flow Immunoassay Rapid RT-PCR, Cotton Tipped Swab, Visually Read	Nasal Swab, Nasal Mid-turbinate Swab	15–30/15–30	95.8, 100
** < 30** FDA EUA https://www.fda.gov/media/136522/download	ID NOW COVID-19 Abbott Diagnostics Scarborough, Inc https://www.abbott.com/IDNOW.html Isothermal Nucleic Acid Amplification Technology Molecular NAAT Test, Foam tipped swabs, Machine Read	Nasal Swab, Nasopharyngeal or Throat Swabs	2–30/15- 30	95.0, 97.9
**30** FDA EUA https://www.fda.gov/media/144253/download	Clip COVID-19 Rapid Antigen Test Luminostics, Inc https://luminostics.com Lateral Flow Immunoluminescent Assay Antigen Test, Cotton-tipped Swab, Machine Read	Anterior Nasal Swab	15- 30/15- 30	96.9, 100
** > 24 hours**^**a**^ https://www.accessdata.fda.gov/cdrh_docs/pdf20/DEN200031.pdf	BioFire Respiratory Panel 2.1 BioMérieux, Inc https://www.biofiredx.com/products/the-filmarray-panels/filmarrayrp/ PCR-based multiplex Nucleic Acid Amplification Technology, Molecular NAAT test, Nylon Flocked Swab, Machine Read	Nasopharyngeal Swab	15–25 (4 h), 2–8 (3 days), ≤ -15 ≤ -70 (30 days)/15–25	97.1, 99.3

a) BioFire has an analysis time of 45 min. However, the specimen collected is shipped overnight to a third-party laboratory to be processed on a BioFire^®^ FilmArray 2.0 system

*CE* Conformité Européenne, *CLIA* Clinical Laboratory Improvement Amendments, *COVID-19* Coronavirus disease 2019, *EUA* Emergency Use Authorization, *FDA* United States Food and Drug Administration, *IgG* immunoglobulin G, *IgM* immunoglobulin M, *NAAT* nucleic acid amplification test, *NP* nasopharyngeal, *NS* nasal swab, *PCR* polymerase chain reaction; and *SARS-CoV-2* severe acute respiratory syndrome Coronavirus 2

**Fig. 8:**
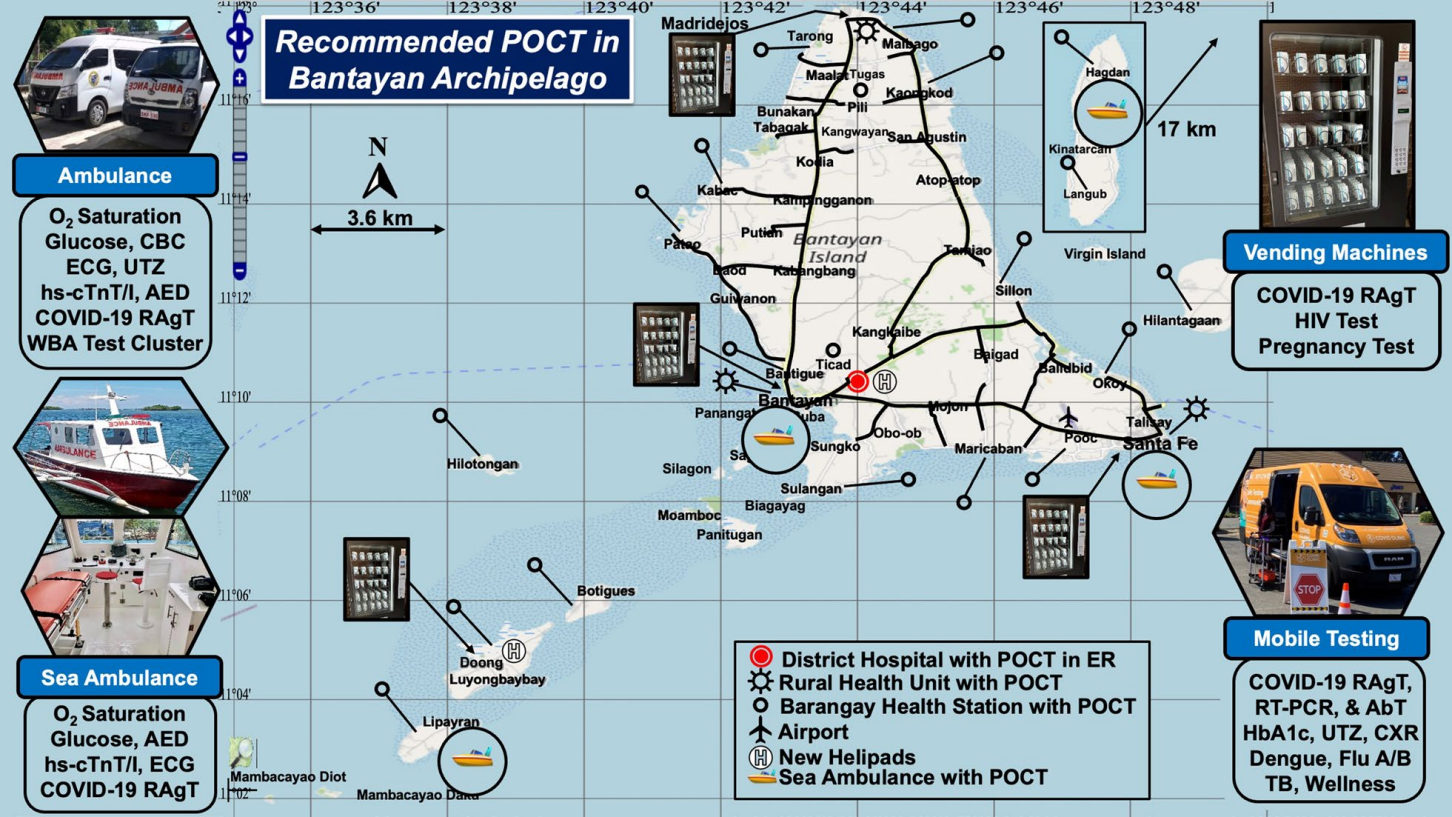
Proposed Sites and Recommended POCT in the Bantayan Archipelago. The POC test clusters shown in the margins draw on outcomes evidence documented in the Compendium and include prehospital tests recommended in Table 3, as well as a third POCT-equipped sea ambulance to be stationed at the Bantayan Port. *AED* automated external defibrillator, *AbT* antibody test, *CBC* complete blood count, *CXR* chest x-ray, *ECG* electrocardiogram, *Flu A/B* influenza A/B, *HbA1c* hemoglobin A1c, *HIV* human immunodeficiency virus, *hs-cTn T/I* cardiac biomarkers troponin T or I, *RAgT*, rapid antigen test, *RT-PCR* reverse transcriptase polymerase chain reaction, *TB* tuberculosis, *UTZ* ultrasound; and WBA, whole-blood analyzer

Figure 8 shows recommended locations for POC diagnostics in the Bantayan Archipelago. These will (a) improve access where natural terrain and interisland distances slow ocean rescue, (b) accommodate people in need of flexibility during increasingly inclement weather and ocean swells, (c) address endemic infectious diseases and new threats, and (d) alleviate spatial injustice in the delivery of urgent care especially during weather crises. We recommend distributing COVID-19 testing to mitigate spread, obviate the need for lockdowns, and protect healthcare personnel [84]. Test kit distribution by vending machines provides several advantages, including easy local access, quick results, and safety [85]. Experience in Canada demonstrated the feasibility of POCT on ambulances [86, 87].

We recommend accelerating intervention for patients with acute myocardial infarction by implementing POC cardiac biomarker testing in ambulances upstream on spatial care paths. Tideman et al. [88] showed that prehospital POC cardiac troponin testing, ECG, and cardiologist guidance during air transport decreased inequities in mortality rates, which were higher for people living in Australian rural versus urban areas; Arnold et al. [89] recommended remote POC services for high risk COVID-19 patients in a consensus statement for both Australia and New Zealand. Spatial care paths for acute myocardial infarction can be designed for other islands in the Visayas, such as the highly linear and remote Palawan Island (please see Fig. 9 in reference [51]).

Point-of-care and prehospital testing are inherently spatial and intrinsically temporal, that is, fast. They can meet several geospatial needs identified in the Bantayan Archipelago, facilitate rapid diagnosis on spatial care paths, and expedite medical decision making for people forced to relocate during ocean encroachment and storm surge flooding. Timely mobility of diagnostics will improve emergency responses, alleviate disparities in geographic access, help avoid future losses, and prepare island communities for more frequent and severe climate change crises.

### Supplementary Information

**Additional file 1.** Compendium of Prehospital Diagnostic Testing and Outcomes.

**Additional file 2.** Collection Tool for Needs Assessment Data.

## Data Availability

Data are available upon reasonable request.

## Abbreviations

BNP/NT-proBNP: Brain natriuretic protein
COVID-19: Coronavirus infectious disease 2019
CTU: Cebu Technological University
CXR: Chest e-ray
DRRMO: Disaster Risk Reduction and Management Office
ECG: Electrocardiogram
EMS: Emergency medical services
HbA1c: Hemoglobin A1c
POCT: Point-of-care testing
RAgT: Rapid antigen test
RT-PCR: Reverse transcriptase polymerase chain reaction
SARS-CoV-2: Severe acute respiratory syndrome-Coronavirus-2
SWN: Small-world network
TB: Tuberculosis
WHO: World Health Organization
