# Supplementary material for: Using geographic rescue time contours, point-of-care strategies, and spatial care paths to prepare island communities for global warming, rising oceans, and weather disasters
Source: Int J Health Geogr. 2023 Dec 20;22:38. doi: 10.1186/s12942-023-00359-y (PMC10731708; doi:10.1186/s12942-023-00359-y)
Supplement: Supplementary file 1 — Additional file 1. Compendium of Prehospital Diagnostic Testing and Outcomes. [file 12942_2023_359_MOESM1_ESM.docx]

***Supplement 1***

***Compendium of Prehospital Diagnostic Testing and Outcomes***

| **Author, Journal, Year (Reference No.)** | **Geospatial Diagnostic Portals** | **Settings/**  **Environment** | **Decision Making**  **Pivots** | **Innovations, Effectiveness, and**  **Impact on Outcomes** |
| --- | --- | --- | --- | --- |
| **Part 1. Strategies for Point-of-need Testing** | | | | |
| **• Ambulances and Emergency Medical Services** | | | | |
| Baker  Aust J Rural Health  2022 [1] | Emergency workforce and resources access planning | Rural Australia | POCT | POCT and clinician radiography were more common in smaller facilities. Small and remote facilities adapt by using different workforce structures and bedside investigations. |
| Fuzery  Clin Lab News  2021 [2] | Ambulance and helicopter EMS teams | Alberta, Canada | POCT standards of care for ambulances | Patients depend on reliable testing form air and ground ambulance teams and the complexity of these environments deserves special considerations leading to standards of care. |
| Fuzery  Arch Path Lab Med  2020 [3] | Ambulance and helicopter EMS teams | Alberta, Canada | POCT in four field settings | POCT in EMS programs should incorporate specific guidance on quality standards that are needed to address the unique challenges of performing POCT in field settings. |
| Heaney  J Paramed Pract  2020 [4] | Prehospital triage using “Labkit” with several POC instruments and several tests | Surrey, London, England (unique empowerment study) | Cobas h232  HemoCue i-Stat StatStrip CoaguChek  Pocket-chem | POCT results available at the time of paramedic assessment reduced ED conveyance by 21%. 10% had POCT results requiring unrecognized admission for urgent Rx. 31% of conveyance decisions were changed by POCT. Results suggest both medical and economic benefits in the prehospital setting. |
| Sanko S  Prehosp Emer Care  2020 [5] | Advance provider response unit in field with POCT | Los Angeles Fire Department, California, USA | POCT | Advanced practice providers such as nurse  practitioners with POCT can be incorporated into the prehospital setting to address a  growing subset of 911-patients whose needs can be met outside of the ED. |
| **• COVID-19 Test Optimization** | | | | |
| Eng  Preprints  2022 [6]  ODH  2021 [7] | Geospatial maps of testing sites, mobile van for POC COVID-19 testing | Rural Cambodian provinces and California, USA | COVID-19 RAgT, RT-PCR, and Antibody test | Strategic placement of field RAgTs and referral RT-PCR enables proper triage and diagnosis of COVID-19 in the rural Cambodian provinces. A mobile van in California provides community access to rapid COVID-19 testing where outbreaks occur. |
| Kost  Diagnostics  2022 [8]  APLM  2019-22 [9-15] | Diagnostic gateways empowering people for self-care | Mathematical analysis of clinical studies, worldwide | COVID-19 RAgT, saliva RT-PCR test | Visual logistics established the relative merits of home, community, and emergency COVID-19 diagnostic portals along spatial care paths that empower people to protect their families, friends, and workplace colleagues. |
| Yolo County Public Health and CDC  2022 [16,17] | Vending machines for COVID-19 test kit free distribution | Yolo County public libraries (4), California, USA | COVID-19 RAgT self-testing kits  (Flowflex) | First county in California where public health distributes COVID-19 RAgTs free of charge in unlimited numbers creating responsibility for frequency of use and 24/7 empowerment when individual results become available in minutes. |
| Clinical outreach pioneers  Investigative reports  2020-2022 [18-26] | Mobile and door-to-door testing | Several sites worldwide | COVID-19 testing outreach at points of need in the community | Benefits comprise high volume testing; improved access; privacy; multiple test formats (PCR, RAgT, AbT); service for hard-to-reach, marginalized, vulnerable, and underserved people diminishing disparities and inequities; increased testing; tracking positivity rates; outbreak mitigation; and partnered vaccination. |
| **• Spatial Care Paths** | | | | |
| Kost  Point of Care 2016-18 [30-32]  Clin Lab Int 2015 [29]  Am J Dis Med 2015 [28]  JIFCCLM 2014 [27] | The spatial care path concept — Invention, construction, and geospatial design | Global applications (see references) | Optimizing patient access and medical decision making | POC testing in limited-resource settings is best guided by thorough understanding of the POC geospacer and culture. The spatial care path facilitates an essential balance of prevention and intervention in public health and shifts future focus to the patient, empowerment, and primary care within the context of geography, topography, healthcare, and culture. |
| Kost  Front Publ Hlth 2019 [36]  Ferguson  Int J Hlth Geogr  2016 [35], GPOC 2015 [34], Point of Care 2012 [33] | Geographic information systems and geospatial analysis | Isaan, Thailand | POC access to cardiac biomarker testing in spatial care paths | Geospatial analyses improve alternative POCT placement in limited-resource settings by revealing deficiencies in health care access. GISs provide a platform for comparing relative costs, assessing benefits, and improving outcomes. This approach can be implemented effectively to enhance cardiac care. |
| **Part 2. Test Categories** | | | | |
| **• Cardiac Biomarkers** | | | | |
| Castro-Portillo Int J Cardiol  2022 [37] | Adults with acute disease transferred to EDs | Valladolid, Spain | NT-proBNP | POCT NT-proBNP predicts early  mortality in acute cardiovascular disease, excellently in cases of syncope, but does not appear to be useful for predicting outcome in patients with acute heart failure. |
| Jones  J Paramed Pract  2022 [38] | Prehospital stratification of chest pain | Bradford, England | HEART pathway (review) | The use of HEART scores or hs-cTn prehospital by paramedics has the potential to improve patient outcomes. |
| Pavlovsky  Prehosp Dis Med  2022 [39] | Prehospital risk stratification of non-ST-segment elevation myocardial infarction | Paris Region, Frances | hs-cTnI [3.2 to 50,000 and detection  limit 1.1 to 1.9 ng/L] (i-Stat 1), ECG | POC cTnI with ECG allow risk stratification of NSTEMI patients and an optimal referral  pathway. Low risk patients should be referred to the ED while high or very high risk, to the  CCU or PCI center. Positive cTnI was associated with high- or very high-risk stratification regardless of the time to onset of pain (P <.0001). |
| Cooper  J Am Coll Cardiol  2021 [40] | AMI triage and rule out in the prehospital setting | Aberdeen, Scotland (secondary analysis of prospective cohort) | hs-cTnI (remainder sample, not POC), ECG | The FN rate of a hs-cTnI value below the LOD and an optimized rule-out threshold missed up to 1 in 40 and 1 in 10 patients with AMI or cardiac death, respectively. Limiting this approach to later presenters or those without known IHD may improve performance. |
| Cooper  Ann Emerg Med  2021 [41] | Prehospital AMI rule out using the HEART score | Aberdeen, Scotland | ECG, cTnI (Samsung LABGEO) | Paramedics use the HEART score to discriminate risk. Even in combination with out-of-hospital POC cTnI testing, the HEART score does not safely rule out major adverse cardiac events. |
| Koper  Neth Heart J  2021[42] | Emergency department, HEART score | Venlo, Netherlands | Fingerstick capillary hs-cTnI | The sensitivity and negative predictive value of the HEART score were 97.0% and 97.6%, respectively, encouraging prospective study of fingerstick POC hs-cTn in a prehospital setting. |
| Lee  Biosensors  2021 [43] | Prehospital electrochemical biosensors | Critical Care and Universities, Taiwan | cTnI | Biosensor results correlated with standard clinical laboratory cTnI, suggesting it can deliver fast and accurate cTnI in prehospital settings for rapid Dx and patient management. |
| Martin-Rodriquez  Eur J Clin Invest  2021 [44] | Prognostic accuracy of  prehospital POC cTnT | Multicentric ambulance-based study, Spain | Cardiac troponin T (cTnT) | Determination of cTnT on the ambulance can help to stratify the risk of patients and to detect unknown early clinical deterioration. In the high-risk group (cTnI >100 ng/L), the mortality rate was 61.7%, and in the low-risk group (<40 ng/L), 2.3%. |
| Stopyra  PloS ONE  2020 [45] | Prehospital stratification of chest pain without evidence of STEMI on ECG | North Carolina, USA | HEART pathway and cTn (i-Stat) | >150 paramedics were trained to use POC cTn and to calculate a HEART score. Structured prehospital risk assessment with POC cTn facilitates early identification of high-risk patients who benefit from rapid Rx and triage to tertiary care facilities with interventional cardiac catheterization capability. |
| Stopyra  Prehosp Emer Care  2020 [46] | Paramedic POC testing when transporting patients | [ECG ST-elevation patients excluded] | Cardiac troponin I (i-Stat) | The specificity and PPV of the POC cTn were 99.2% and 85.7%; sensitivity and NPV, 26.5% and 87.5%. Prehospital cTn can rule-in AMI but should not be used to exclude AMI because of low sensitivity. |
| Alghamdi  Prehosp Dis Med 2020 [47]  Emer Med J 2018 [48] | Prehospital rule out of AMI (vs. rule in) | Manchester, England | Cardiac troponin (review paper) | Current evidence does not support the use of POC troponin to exclude AMI due to issues with diagnostic accuracy and insufficient high-quality evidence. |
| Harjola  ESC Heart Failure  2020 [49] | Multicenter survey of prehospital protocols for AHF | Belgium, Finland, France, Spain, USA, Switzerland | cTn, BNP, 12-lead ECG, UTZ | UTZ and POCT were available in advanced life support and helicopter EMS units in <25% of EMS regions. Protocols for ST-elevation AMI, chest pain, and dyspnea were present in 95.2, 80.8, and 76.0% of EMS regions, respectively. |
| Johannessen  Open Heart  2020 [50] | Prehospital primary care emergency clinic | Oslo, Norway  (low prevalence) | hs-cTnT in 1 hour algorithm | hs-cTnT seems safe, efficient, and applicable for accelerated assessment of patients with non-specific chest pain. Sensitivity was 98.4%; NPV, 99.9%; and 90-day rule out morality; 0.3%. |
| Kaier  J Am Heart Assoc  2019 [51] | Blood samples from ambulance paramedics | England, Denmark, and Germany | Cardiac myosin-binding protein C | Very early after symptom onset, cardiac myosin-binding protein C improves diagnostic discrimination and could significantly improve the early triage of patients with suspected AMI. |
| Alghamdi  Emerg Med J  2018 [52] | Prehospital rule out of ACS | Manchester, United Kingdom | cTn (a review of 9 papers) | Based on the currently available evidence,  POC troponin assays are insufficiently sensitive to rule out ACS in the prehospital environment. |
| Kost  Point of Care  2018 [32] | Designs for AMI spatial care paths | Central Vietnam | Cardiac biomarkers | Early upstream POC cTn testing on spatial care paths will expedite transfers directly to hospitals capable of intervening, improving decision making and outcomes following coronary occlusion. |
| Rasmussen  EHJACC  2017 [53] | Prehospital diagnosis and risk stratification | Aarhus, Denmark | cTnT | Patients presenting cTn T ≥50 ng/L had 24% 1-year mortality compared with 4.8% for cTnT <50 ng/L (p<0.001). Prehospital cTnT ≥50 ng/L has a poor prognosis irrespective of final diagnosis and high predictive value for identifying high-risk prehospital so that they may be rerouted directly for advanced care at an invasive center. |
| Stengaard  Biomarkers  2016 [54] | Optimizing prehospital triage | Aarhus, Denmark | hs-cTnT,  copeptin in prehospital samples | hs-cTnT and copeptin performed prehospital could potentially improve the diagnostic and prognostic classification of patients with suspected AMI (not POC). |
| Ezekowitz  J Am Heart Assoc  2015 [55] | Prehospital cTn testing in ambulances | Alberta, Canada | cTn | Ambulance cTn accelerated the time to final disposition. Enhanced and more cost-effective early ED discharge of most patients with chest pain is an unrealized opportunity. |
| Tideman  Med J Aust  2014 [56] | Royal Air Service fixed wing air ambulance | Rural Australia | POC cTn | Cardiologist-supported remote risk stratification, management, and facilitated access to tertiary hospital-based early invasive management are associated with improved 30-day mortality for patients who initially present to rural hospitals and are diagnosed with AMI. These interventions closed the gap in mortality between rural and metropolitan patients in South Australia. |
| Stengaard  Am J Cardiol  2013 [57] | Prehospital triage | Aarhus, Central Denmark | Quantitative cTnT | Quantitative prehospital cTnT testing by paramedics is feasible and an elevated cTnT is highly predictive of mortality in suspected AMI. |
| Venturini  Prehosp Emer Care  2013 [58] | Testing in moving ambulances versus the ED | Maywood, Illinois, USA | cTnI testing site comparison study | No significant difference in whole-blood troponin results was found between cTnI performed in the moving ambulance and the ED. |
| Prosen  Crit Care 2011 [59] | Differential Dx in prehospital emergencies | Maribor, Slovenia | NT-proBNP, UTZ | UTZ comet-tail sign alone or in combination with NT-proBNP has high diagnostic accuracy in differentiating acute HF-related from  COPD/asthma-related causes of acute dyspnea. |
| Sorensen  Am J Cardiol  2011 [60] | Prehospital Dx and triage of AMI | Aarhus, Denmark | Qualitative cTnT, ECG | Prehospital cTnT testing is feasible with a high success rate and indicates that implementation of quantitative tests with lower detection limits could identify most patients with AMI  irrespective of ECG changes. |
| Kost  Point of Care  2010 [61] | Implementation of POC cTn testing in rural provinces | Thailand | cTnT, NT-pro-BNP | Following defining pilot studies of need and application, cobas 232 handheld cardiac biomarker testing was implemented in hundreds of sites transforming the care of patients with AMI. |
| Di Serio  Clin Chem Lab Med  2006 [62] | Ambulance tele-cardiology for managing AMI | Bari, Italy | cTnI (i-Stat), ECG (no patients with ST-segment elevation) | For POCT, cTnI was >0.09 microg/L in 20 AMI patients (91%). The median ambulance TAT was 12 min and median hospital TAT, 40 min. cTnI integrated with tele-medicine plays an important role in management of ACS patients related to the prehospital phase early Dx/Rx. |
| Owens  J Electrocardiol  2004 [63] | Physician-manned mobile CCU | Belfast, Northern Ireland | 80-lead ECG body map | The 80-lead BSM is superior to the standard 12-lead ECG in predicting AMI prehospital. |
| Schuchert  Am Heart J  1999 [64] | Ambulance cTnT-6-mo follow-up of AMI and death | Hamburg, Germany | Qualitative cTnT | Rapid cTnT identified a minority of patients with AMI. Positive prehospital TnT was an objective marker for a worse outcome. During follow-up, patients positive prehospital for cTnT had cardiac events more often (9 of 11) than patients with a negative result (26 of 147; P <.0001). |
| **• CRP** | | | | |
| Matthes A  BMJ Open  2023 [65] | Outpatient emergency medical services | Jena, Germany | POC CRP | Quantitative POC CRP promotes step-down clinical decisions and strengthens the clinical confidence of physicians in out-of-hours outpatient emergency medical services. |
| **• Electrolytes, Blood Gases, Ca^++^, and pH** | | | | |
| Collopy  Air Med J  2022 [66] | POCT during ground & helicopter transport | Wilmington, North Carolina, USA | pO_2_, pCO_2_, pH, Na^+^, K^+^, Ca^++^, Glu, Cr, Lactate, Hct, [epoc] | Care plan alterations occurred in 38.6% of patients undergoing POCT, which most frequently changed care for post-cardiac arrest syndrome (64.7%), sepsis/septic shock (61.8%), diabetic ketoacidosis (54.5%), and pneumonia (49.3%). |
| Morton  Air Med J  2022 [67] | Prehospital helicopter emergency services | England | Arterial BG, lines (review) | Prehospital guidelines state that monitoring should match in-hospital standards. Clinicians  believed arterial BG would allow better  monitoring and more targeted treatment. |
| Murali  Clin Chem Lab Med  2022 [68] | Supporting environment-al stability during air transport | Pittsburgh, Pennsylvania, USA | BG | Temperature excursions are reduced by insulated transport bags with heating and cooling packs. POC blood gas results during air transport improved ventilator management, increased recognition of ventilation-perfusion mismatch, and improved patient tolerance of ventilation. |
| Cini  Recenti Prog Med  2021 [69] | Home evaluation of COVID-19 patients | Toscana Sud Est., Italy | Potable blood gas analyzer | POCT blood gas analysis made it possible to evaluate and treat at home 52% of COVID-19 patients and admit others directly to the most appropriate ward. |
| Gruebl  SJTREM  2021 [70] | Prehospital Dx and Rx for out-of-hospital cardiac arrest | Essen, Netherlands | pO_2_, pCO_2_, pH, Na^+^, K^+^, Ca^++^, glucose, lactate, Hct, Cr [epoc] | Prehospital POCT allows rapid detection of pathological acid-base imbalances and potassium and specific interventions that improve the probability of survival. 21% of patients survived to hospital discharge (with POCT, 30%, versus no POCT, 16%, p = 0.01). |
| Nawrocki  Front Physiol  2021 [71] | High altitude hospital (760 m) and clinic (3,100 m) | Zurich, Switzerland | Portable blood gas analyzer (epoc) | Because of portability and ease of handling, portable blood gas analyzers are valuable diagnostic tools for use in everyday  practice as well as under challenging field conditions at high altitudes. |
| McPherson  J Paramed Pract  2019 [72] | Prehospital patient disposition | So. Central Ambulance, England | VBG, urea, lactate, Hct, Hgb, electrolytes | POCT improved clinician confidence in decision making and patient disposition validated by better discharge on scene and recontact rates. POCT knowledge increased. |
| Shin  Crit Care  2017 [73] | Out of hospital cardiac arrest with ER Rx | Seoul and multicenter sites, South Korea | pH, K^+^, and other ER POC tests | pH and K^+^ levels were independent factors associated with survival to hospital discharge, and pH level was an independent factor related to neurological recovery. |
| Mikkelsen  SJTREM  2015 [74] | Prehospital blood gas, pH, and metabolite analysis in a mobile emergency care unit | Odense, Denmark | WBA (Radiometer ABL-90) | Only minute changes were needed to use the ABL-90, which helped direct Rx of COPD; release of patients with suspected CO poisoning; speed Dx, blood cultures, and antibiotic Rx of septic shock and septicemia; obviate cyanide toxicity/ anaerobic metabolism from fire exposure through determining lactate levels; and adjust respiratory parameters for intubated patients with elevated intracranial pressure, for which BG pCO_2_ fine tuning excelled management using end-tidal CO_2_. |
| Di Serio  Clin Chem Lab Med  2010 [75] | Performing SWOT analysis for air ambulances | Bari, Italy | Arterial BG, glucose, Na^+^, K^+^, Ca^++^, Hct/ Hgb | Clinical study results suggest that critical tests performed during the transport of critically ill patients improve patient care. Real-time results during transport must be considered an integral part of the patient care process. Excellent channels of communication are needed between the ICUs, EMS, and laboratories. |
| Jousi  World J Emer Surg 2010 [76] | Trauma resuscitation with POCT at accident site | Helsinki, Finland | BG | A portable BG analyzer was useful in prehospital monitoring for trauma resuscitation and led to significantly greater awareness of base excess and pH values. |
| Vos  World J Emerg Surg  2010 [77] | POCT during interhospital transport of critically ill pediatric patients | Maastricht, Netherlands | BG, K^+^, Hct, others | POCT led to 42.9% of therapeutic interventions for life threatening results that could not have been discovered otherwise. We recommend POC BG, electrolytes, glucose, and hematocrit during interhospital transports, especially for mechanical ventilator adjustment. |
| Gruszecki  Clin Chem  2003 [78] | Critical care transport in ambulances and twin-engine jets | Birmingham Alabama, USA | pO_2_, pCO_2_, pH, TCO_2_, O_2_ Sat, Na^+^, K^+^, Cl^-^, urea, Cr, Hct, glucose | Our experience shows that POCT can be performed with high reliability and provides rapid critical analyte and blood gas results where no other laboratory analysis is available. POCT in the transport setting is infrequent and is relatively inexpensive. Use of POCT led to changes in patient treatment 30% of the times when testing was performed. |
| Backer  S Ann Emerg Med  1999 [79] | Back-country evaluation of heat symptoms | Grand Canyon National Park, USA | Na^+^ (i-Stat) | POC Na^+^ reliably identified exercise-associated hyponatremia, an important cause of heat illness during endurance exercise. The results helped make Rx and disposition decisions. |
| Herr  Am J Clin Path  1995 [80] | Performing prehospital Dx and Rx on helicopters | Rochester, Minnesota, USA | Na^+^, K^+^, Hct, Hgb (i-Stat) | Fifteen (18.5%) patients were treated with transfusions, glucose, or insulin based on results obtained during helicopter flight. |
| **• Glucose and Lactate** | | | | |
| Fuzery  Clin Lab News  2021 [2]  Arch Path Lab Med  2020 [3] | Ambulance preparedness and protocols | Alberta Health Services, Canada | Glucose | All ground ambulances operated by AHS have glucose meters, which can be operated by paramedics trained in basic life support only. Paramedics follow written protocols if they suspect hyper/hypoglycemia. The protocols provide direction on when to do a glucose meter test and how to act on the result. |
| Hill  Air Med J  2020 [81] | Air ambulances in Midwest America | Colorado & Ohio, USA  [nondiabetic patients] | Glucose | POC glucose greater than 220 mg/dL should prompt prehospital aggressive balanced resuscitation before arrival at the trauma center to prevent worsening hypotension and hemorrhagic shock. |
| Remick  Prehosp Emer Care  2017 [82] | Assessing prehospital seizures in pediatric patients | Harbor-UCLA Hospital, California, USA | Glucose | Findings suggest the importance assessing  prehospital seizure protocols, which should be studied to identify unique cases where glucose testing might be useful in view of the rarity of prehospital seizures in pediatric patients. |
| Lerner  Am J Emerg Med  2003 [83] | Paramedic decision making in the field | Buffalo, New York, USA | Glucose | Paramedics successfully treated, without complication, most of the patients with uncomplicated hypoglycemic events. Patients preferred discharge without transport to an ED. |
| Novak  J Paramed Pract  2022 [84] | Rapid response vehicle with POCT | Oxford, England | Lactate, pH, TCO_2_, Ca^++^, Cr,  others (i-Stat) | POCT by ambulance services is feasible and when combined with telephone advice and decision support from physicians, may be effective in reducing hospital admissions for frail patients in supportive care environments. |
| Galvagno  Shock  2020 [85] | Helicopter prehospital Dx and intervention | Baltimore, Maryland, USA | Lactate (i-Stat) | Lactate outperformed vital signs, including shock index, for detecting shock and predicting the need for LSIs. A lactate level > 4 mmol/L was highly associated with need for LSIs. |
| Martin-Rodriquez  Eur J Clin Invest  2020 [86] | Prehospital POCT on ambulances | Valladolid, Spain | Lactate | 4 mmol/L was the cutoff for low versus high mortality, highlighting the importance of lactate to determine risk of early in-hospital mortality. EMS lactate could improve identification of risky patients and better care. |
| Martin-Rodriquez  Am J Emer Med 2018 [87] | Prehospital prediction of early (< 30 d) mortality | University of Valladolid, Spain | Lactate | POC lactate with the best sensitivity (84%) and specificity (70%) overall was 4.25 mmol/L. The level can guide us early in the detection of critical patients. |
| **• Hemoglobin A1c** | | | | |
| Krass  Diabetes Res Clin Pract  2023 [88] | Pharamacy-based screening for type 2 diabetes | Multicenter, Australia | HbA1c, risk assessment, and capillary glucose | In community pharmacies, the most effective method to uncover undiagnosed T2DM was a stepwise approach — initial risk assessment and if  appropriate an HbA1C POC test and referral. |
| Ventura  Point of Care  2019 [89] | Management of prediabetes and diabetes in community health centers | Rural Central Vietnam | HbA1c | HbA1c and blood glucose instruments should be available; public health must fund self-monitoring, glucose meters, and enhanced access; and diagnostic cutoffs for HbA1c should be harmonized after checking population differences. |
| Shephard  Clin Biochem  2017 [90] | National rural indigenous POCT program for diabetes | Rural Australia | HbA1c and quality control | HbA1c POCT in the Aboriginal and Torres Strait Islander Medical Services program has remained analytically sound, matched the quality achieved by Australasian laboratories, and met profession-derived analytical goals for 15years. |
| Motta  Primary Care Diabetes  2017 [91] | Primary care delivery | South Africa | HbA1c | POCT changed clinical practice by facilitating access to HbA1c testing. Patients achieving optimal glycemic control (HbA1c ≤ 6.5-7.5%) increased by 125%, while those with very poor control (HbA1c > 10%) halved. Mean HbA1c at the first POC test decreased from 9.7% ± 2.4 to 8.4% ± 2.4 for the most recent (paired t-test p<0.01). |
| Kost  Point of Care  2017 [31]  2011 [92] | Rotating POCT in several rural villages, drones for specimen transport | Limited-resource  provinces in Isan and northwest Thailand | HbA1c | Rapid on-site HbA1c testing efficiently identified those poorly controlled. Elevated HbA1c changed primary care strategy, pulling together a rotating team of physicians, nurses, and a pharmacist who adjust therapy and check for albuminuria to prevent advancing disease, dialysis, and adverse outcomes. |
| Spaeth  Rural Remote Health  2014 [93] | POC HbA1c screening and monitoring | Indigenous populations in remote Australia | HbA1c | We demonstrated POCT can improve timeliness and clinical follow-up in remote locations, while also reinforcing clinical and cultural effectiveness in assisting to improve diabetes management in Indigenous Australians. |
| Martin  Med J Aust  2005 [94]  Shephard  Rural Remote Health  2005 [95] | Managing diabetes along the rural Mallee Track — top 10 health problems | Building Health Communities, Victoria State, Australia | HbA1c | POC capillary HbA1c testing offers an accurate, practical, and community-friendly way of monitoring diabetes in rural and remote clinical settings. All community and health professional groups surveyed agreed that the POC model should be available to all rural people. |
| **• Hemostasis and D-dimer** | | | | |
| Schober  Am J Emer Med  2021 [96] | Helicopter feasibility decision making | Amsterdam, Netherlands | PT and INR (i-Stat) | Coagulopathy in trauma (e.g., coagulation factor consumption) and nontrauma cases (e.g.  anticoagulant) demand PT/INR results which were proven feasible in helicopter EMS. |
| Beynon  SJTREM  2015 97] | Prehospital hemostasis monitoring | Heidelberg, Germany | PT, INR | Assessment of INR in prehospital emergency care provides valuable information on hemostatic parameters in patients. |
| Rumpf  Crit Care  2006 [98] | Dx of PE in prehospital emergency setting | Maribor, Slovenia | D-dimer, PetCO_2_ | For PE confirmation, PetCO_2_ had sensitivity 92.6%, NPV 94.2%, specificity 83%, and PPV 79.2%. Combined clinical probability and PetCO_2_ may safely rule out PE in patients with suspected PE and positive D-dimer prehospital. |
| **• Ultrasound Imaging** | | | | |
| Christenson  SJTREM  2022 [99] | Home COPD POC intervention | Odense, Denmark | Ultrasound and blood analysis | Emergency medical technicians must show experience and safety in handling shortness  of breath as well as POC diagnostics. |
| Ienghong  Prehosp Dis Med  2022 [100] | Prehospital  EMS for Srinagarind Hospital | Khon Kaen, Thailand | Handheld POC ultrasound | Performed on the lung (37.0%), inferior vena cava (30.8%), and cardiac cases (26.4%); 34.9% abnormal and 66 cases (39.1%) with diagnoses confirmed and an accuracy peak of 75.8%. |
| Dubecq  J Trauma Acute Care Surg  2021 [101] | Triage,  diagnosis, severity, Rx,  and priority for surgery | French military austere combat settings | Ultrasound-Africa, the Middle East | Ultrasound is valuable for management of mass casualties by improving treatment and triage, especially when surgical resources are  limited and can also correct a diagnosis or improve prehospital therapeutic choices. |
| Nadim  BMC Health Serv Res  2021 [102] | Timely release of patients at the scene | Odense, Denmark | Ultrasound and blood analysis | Prehospital emergency medical technicians can perform UTZ and blood analysis in prehospital evaluation of patients with COPD. None released requested a secondary ambulance within the first 48 hours following intervention. |
| Schoeneck  West J Emer Med  2021 [103] | Prehospital Dx of CHF, pulmonary edema | New Haven, Connecticut, USA | Portable thoracic ultrasound | Prehospital lung ultrasound B-lines may aid in identifying or excluding CHF as a cause of dyspnea and has reasonably sensitive and specific for the diagnosis of CHF and pulmonary edema. |
| Sabatino  J Ultrasound  2020 [104] | Primary imaging modality | Sierra Leone, Africa | POC ultrasound | POC UTZ represents a powerful diagnostic tool in a low-income country to improve patient management. Training of nonphysician health providers is doable to improve healthcare in resource-limited settings. |
| Bobbia  Anaesth Crit Care Pain Med  2018 [105] | Dx in EDs and mobile intensive care stations | Nimes, France | Ultrasound | Almost 75% of EDs and nearly 1/3 of mobile intensive care stations are equipped with at least one UTZ device. Physicians trained remains insufficient. UTZ was used at least three times a day in 41% of EDs and 19% of mobile stations. |
| Chin  J Emer Med  2013 [106] | Field Dx using UTZ | Houston, Texas, USA | Ultrasound | UTZ field diagnoses of pneumothorax, pericardial effusion, or cardiac standstill may directly impact patient resuscitation in the field. |
| **• Toxicology** | | | | |
| Advance Health Medical Cooperative 2022 [107] | Mobile van testing and medical examination | Cebu City, Visayas, Philippines | Drug and chemistry tests | Provides mobile diagnostic clinic and testing services in the community for Cebu City and surrounding regions. |
| Soderqvist  Point of Care  2018 [108] | Prehospital drug testing for mobile ICU patients | Tampere, Finland | Drugs of abuse | Oral fluid screening for illicit substances can be a valuable diagnostic tool in addition to the usual diagnostic methods in EMS patients with unconsciousness due to an unknown cause or intoxication. |
| **'Part 3. Clinical Problems** | | | | |
| **• Antiplatelet Therapy** | | | | |
| Levens  Genes  2023 [109] | Community pharmacies antiplatelet therapy | Netherlands | CYP2C19 pharmaco-genetic testing | POC CYP2C19-guided de-escalation of prasugrel/ticagrelor to clopidogrel appears feasible in a community pharmacy setting. |
| **• Infectious Diseases** [See **Part 1** for COVID-19 strategies] | | | | |
| Do Nitt  Lancet InfectDis  2023 [110] | POCT in commune health centers | Vietnam | CRP testing in acute respiratory infections | POC CRP testing efficaciously reduced  prescription of antibiotics in patients with nonsevere acute respiratory infections without compromising patient recovery. |
| Mital  AIDS  2023 [111] | POCT in pharmacies, self-testing, and lab testing | Multicenter, Canadian Provinces | HIV | POCT at community pharmacies can generate substantial cost savings and improve health outcomes compared with standard lab testing. It would  also be cost-effective vs. HIV self-testing. |
| Sheehan  J Hepatol  2023 [112] | Inmates in prisons | Australia | POC Hepatitis C RNA testing | POCT enhanced treatment uptake and  reduced time to treatment initiation among people recently incarcerated, thereby overcoming key barriers to treatment in the prison sector. |
| Tinsay S, Public Health Team  “introducing New Tools TB Program”  2022-23 [113] | Tuberculosis peripheral care point of need screening | Bantayan Archipelago, Philippines | Tuberculosis | POCT is cost-effective, requires minimal biosafety requirements, and is intended for the peripheral level, that is, community health facilities or mobile clinics. Pilot program discovered ~9% infected with TB out of 432 patients screened in the Municipality of Bantayan. Screening continues currently. |
| Beeman  Afr J Emer Med  2022 [114] | Patients seeking emergent injury care | Kingali, Rwanda | HIV | Emergency centers are important access points for HIV testing. In Rwanda, 1 in 8 with HIV are unaware of their infection, which impedes epidemic control. This could be addressed by increased testing. |
| Zadran  Diagnostics  2022 [115] | Lifeline of diagnostic entry points | Central Vietnam | POCT for infectious diseases | Infectious disease testing should be improved and POC tests supplied near patients’ homes and in primary care settings for the early detection of infected individuals and the mitigation of the spread of new COVID-19 variants and other highly infectious diseases, such as Monkeypox. |
| WHO  Guidelines on HIV Testing  2021 [116] | Decentralized, stand-alone, and self-testing | General | HIV | Community-based rapid HIV testing in both high- and low-prevalence settings closer to people’s homes and self-testing can reduce transport costs and waiting times in central hospitals and thereby increase uptake. |
| Vallely  Welcome Open Research  2019 [117] | POCT for sexually transmitted infections and immediate Rx | Rural health units in limited-resource  Papua New Guinea | Antenatal POCT for STD | First randomized trial to evaluate the effectiveness, cost-effectiveness, acceptability, and health system requirements of POC STD testing and treatment to improve birth outcomes in high-burden settings by decreasing preterm and low weight births. |
| West K  J Emer Med Serv 2007 [118], 2004 [119] | Protecting prehospital providers | General | HIV | Recommendations for protection of prehospital providers and postexposure testing. |
| **• Sepsis** | | | | |
| Metelmann  Anesthetist  2018 [120] | Prehospital, emergency staff survey about high sepsis mortality | Greifswald, Germany | qSOFA (quick sequential organ failure assessment) | The majority selected “increased body temperature”, “drop in blood pressure” and “altered breathing;” emergency doctors selected “altered mental status” more frequently than nurses and paramedics. All rated early fast sepsis treatment as important. |
| **• Seizures** | | | | |
| Donosos-Calero  Eur J Clin Invest  2023 [121] | Patients presenting with prehospital seizures | Life support units in Spain | RR, pCO_2_, BUN, K^+^, glucose, O_2_ Sat, Hgb | Prehospital variables reflect clinical impairment and mortality of patients suffering from seizures. |
| **• Stroke** | | | | |
| Ebinger  JAMA  2021 [122] | Mobile stroke unit, on-board thrombolysis | Berlin, Germany | Mobile CT scanner and POCT | Dispatch of mobile stroke units, compared with conventional ambulances alone, was associated with lower global disability at three months. |
| Wendt  Stroke  2015 [123] | Prehospital triage of stroke patients | Berlin, Germany | CT scanner with POCT laboratory on board | Triage of patients with cerebrovascular events to specialized hospitals with stroke units and capabilities for ischemic stroke and neurosurgery can be improved by stroke emergency mobile ambulances with POCT. |
| Ebinger  JAMA  2014 [124] | Starting thrombolysis in an ambulance | STEMO Consortium, Berlin, Germany | CT scanner with POCT laboratory on board | Compared with usual care, ambulance-based CT scanner and POCT for Dx of ischemic stroke initiated thrombolysis decreasing time to Rx without an increase in adverse events. |
| Walter  Lancet Neurol  2012 [125] | Mobile stroke unit to accelerate Dx/Rx | Homburg, Germany | CT scanner,  POCT, and  telemedicine | Only 2-5% of patients receive thrombolytic treatment due to delay reaching hospitals. The mobile unit substantially reduced median time from rescue call to Rx decision, solving the problem of the arrival too late at the ED. |
| **Part 4. Healthcare Access** | | | | |
| **• Community Paramedics** | | | | |
| Paramedics’ new roles  [126-129]  Paramedic examples  [130,131] | Paramedicine-augmented health delivery | Community sites | POCT is integral to the community paramedic programs | Paramedicine programs improve access to nonemergent health care in rural and remote communities by allowing paramedics and EM technicians to operate in expanded roles assisting with public health, primary care, and preventive services to underserved populations. Community paramedics can provide and connect patients to primary care services; complete posthospital follow-up care; integrate local public health agencies, home health agencies, and health systems; educate and promote wellness; and offer services not available elsewhere. POCT is a successful and valuable part of these paramedicine programs. |
| **• Hospital at Home** | | | | |
| Hospital at Home Concept [132-137]  Home examples  [138,139] | Continuum care in residences | Home settings | POCT during in-person visits | Hospital-level care in the comfort of the patient’s home when registered with a hospital. Caregivers visit daily supplemented by virtual visits. POCT is used during in-person visits. Blood for hospital laboratory testing may be dropped off at the laboratory by the caregiver after their in-person visit. |
| **• Unmanned Aerial Systems (Drones)** | | | | |
| Investigator  Application Reports  2015-2022  [140-147]  and  Kost  Point of Care  2017 [31] | POC delivery options | Remote settings | Test kits, nucleic acid diagnostics, specimens, blood products | Transporting specimens via small drones does not affect the accuracy of routine chemistry, hematology, and coagulation tests results. Drone-based medical delivery models offer an innovative approach to addressing longstanding issues of health care access and equity and are particularly relevant to COVID-19. Additionally, the use of an AED-equipped drone has the potential to reduce time to defibrillation in out-of-hospital cardiac arrest. |

**Abbreviations:** AbT, antibody test; ACS, acute coronary syndrome; AED, automated external defibrillator; AHF, acute heart failure; AHS, Alberta Health Services; AMI, acute myocardial infarction; *APLM, Archives of Pathology and Laboratory Medicine;* BG, blood gases (pO_2_, pCO_2_, pH); BNP, blood natriuretic peptide (or NT-proBNP); BUN, blood urea nitrogen; Ca^++^, ionized calcium, typically measured in whole blood; CCU, cardiac (coronary) care unit; CHF, congestive heart failure; CO, carbon monoxide; COPD, chronic obstructive pulmonary disease; COVID-19, Coronavirus infectious disease 2019; Cr, creatinine; CRP, C-reactive protein; CT, computerized tomography; cTn, cardiac troponin I (cTnI) or T (cTnT); Dx, diagnosis; *EHJACC, European Heart Journal - Acute Cardiovascular Care*; ED, emergency department; EMS, emergency medical services; ER, emergency room; FN, false negative; HC, healthcare; Hct, hematocrit; HEART, protocol comprising history, electrocardiogram, age, risk factors, and troponin; HF, heart failure; Hgb, hemoglobin; ; hs-cTn, high sensitivity cTn; ICU, intensive care unit; IHD, ischemic heart disease; INR, International Normalized Ratio; *JAMA, Journal of the American Medical Association*; *JCH,* *Journal of Cambodian Health*; *JIFCCLM, Journal of the International Federation of Clinical Chemistry and Laboratory Medicine*; LSI, life-saving interventions; NPV, negative predictive value; NSTEMI, non-ST-segment elevation myocardial infarction; O_2_ Sat, oxygen saturation; *ODH, Omnia Digital Health*; PCI, percutaneous coronary intervention; PE, pulmonary embolism; PetCO_2_, probability and end-tidal CO_2_; POC, point-of-care; POCT, POC testing; PPV, positive predictive value; PT, prothrombin time; Publ Hlth, Public Health; qSOFA, quick sequential (sepsis-related) organ failure assessment; RAgT, rapid antigen test; RR, respiratory rate; RT-PCR, reverse transcriptase-polymerase chain reaction; Rx, treatment; *SJTREM, Scandian Journal of Rescue, Trauma, and Emergency Medicine*; STEMO, stroke emergency mobile (unit); STI, sexually transmitted infections; SWOT, strengths, weaknesses, opportunities, and threats; SWN, small-world network; T2DM, type 2 diabetes; TAT, turnaround time; TB, tuberculosis; TCO_2_, total carbon dioxide; VBG, venous blood gasses; WHO, World Health Organization; and WBA, whole-blood analyzer.

**Supplement 1 References (147)**

1. Baker T, Moore K, Lim J, Papanastasiou C, McCarthy S, Schreve F, et al. Rural emergency care facilities may be adapting to their context: A population-level study of resources and workforce. Aust J Rural Health. 2022;30(3):393-401.
2. Fuzery AK, Kost GJ. Point-of-care testing by ambulance teams: An opportunity for a new standard. Clin Lab News. 2021;47:6,8-13.
3. Fuzery AK, Kost GJ. Point-of-care testing practices, failure modes, and risk mitigation strategies in emergency medical services programs in the Canadian province of Alberta. Arch Path Lab Med. 2020;144:1352-1371.
4. Heaney K, Whiting K, Petley L, Fry I, Newton A. Point-of-care testing by paramedics using a portable laboratory: an evaluation. J Paramed Pract. 2020;12(3);100-108.
5. Sanko S, Kashani S, Ito T, Guggenheim A, Fei S, Eckstein M. Advanced practice providers in the field: Implementation of the Los Angeles Fire Department Advanced Provider Response Unit. Prehosp Emerg Care. 2020;24(5);693-703.
6. Eng M, Kost GJ. Geospatial analysis and novel testing strategies can help build COVID-19 resilience in resource-poor settings. <https://preprints.jmir.org/preprint/47416> Accessed November 22, 2023.
7. Eng M, Zadran A, Kost GJ. COVID-19 risk avoidance and management in limited-resource countries. Point-of-care strategies for Cambodia. Omnia Digital Health. 2021;6/7:112-115.
8. Kost GJ. The Coronavirus Disease 2019 spatial care path: Home, community, and emergency diagnostic portals. Diagnostics. 2022;12(1216):1-16.
9. Kost GJ. The COVID-19 Grand Challenge: Setting expectations and future directions for community and home testing. Arch Path Lab Med. 2022;146:789-790.
10. Kost GJ. Home antigen test recall affects millions: Beware false positives, but also uncertainty and potential false negatives. Arch Path Lab Med. 2022;146:403.
11. Kost GJ. Diagnostic strategies for endemic Coronavirus disease 2019 (COVID-19) — Rapid antigen tests, repeat testing, and prevalence boundaries. Arch Path Lab Med. 2021;146:16-25.
12. Kost GJ. The impact of increasing disease prevalence, false omissions, and diagnostic uncertainty on Coronavirus disease 2019 (COVID-19) test performance. Arch Path Lab Med. 2021;145:797-813.
13. Kost GJ. Designing and interpreting COVID-19 diagnostics: Mathematics, visual logistics, and low prevalence. Arch Path Lab Med. 2021;145:291-307.
14. Kost GJ. Geospatial spread of antimicrobial resistance, bacterial and fungal threats to COVID-19 survival, and point-of-care solutions. Arch Path Lab Med. 2021;145:145-167.
15. Kost GJ. Geospatial hotspots need point-of-care strategies to stop highly infectious outbreaks: Ebola and Coronavirus. Arch Pathol Lab Med. 2020;144:1166–1190.
16. Community vending machines. City of Davis, Public Health Department, Yolo County, California. 2022. <https://cityofdavis.org/coronavirus> Accessed November 22, 2023.
17. Centers for Disease Prevention and Control. ELC Enhancing Detection Expansion. Epidemiology and Laboratory Capacity for Prevention and Control of Emerging Infectious Diseases. Atlanta, GA: CDC. 2021, 17 pages. <https://www.cdc.gov/ncezid/dpei/pdf/elc-enhancing-detection-expansion-faq-508.pdf> Accessed November 22, 2023.
18. Baker DR, Cadet K, Mani S. COVID-19 testing and social determinants of health among disadvantaged Baltimore neighborhoods: A community mobile health clinic outreach model. Popul Health Manag. 2021;24(6): 657-663.
19. Fiscus L, Towns R, Wood S, Oliver P, Fox S, Weathers A, et al. Spotlight on the safety net: Deploying mobile COVID-19 testing programs in North Carolina as an approach to improving health equity. N C Med J. 2021;82(1):80-82.
20. Frimpong M, Amoako YA, Anim KB, Ahor HS, Yeboah R, Arthur J, et al. Diagnostics for COVID-19: A case for field-deployable, rapid molecular tests for community surveillance. Ghana Med J. 2020;54(4 Suppl):71-76.
21. Gulley T, Tyson T, Collins E, Helton R, Hill-Collins P, France N, et al. The Health Wagon Partners with the Virginia Department of Health to provide COVID-19 testing in rural Southwest Virginia. J Appalach Health. 2020;2(3):146-149.
22. Jiménez J, Parra YJ, Murphy K, Chen AN, Cook A, Watkins J, et al. Community-informed mobile COVID-19 testing model to addressing health inequities. J Public Health Manag Pract. 2022;28(Suppl 1):S101-s10.
23. Lau CSM, Johns J, Merlene S, Kanya S, Taber A, Melander D, et al. Trends in COVID-19 testing and positivity rates from a mobile testing program in the Phoenix metropolitan area. J Community Health. 2021;46(6):1221-5.
24. Lau CSM, Shu S, Mayer J, Towns M, Farris A, Washington F, et al. COVID-19 trends in the Phoenix metropolitan area from a mobile testing program: Last quarter of 2020. J Community Health. 2021;46(6):1078-82.
25. Neely J, Eddins A, Lesure N, Dee D, Real R, Singer R, et al. Privacy, or the lack thereof, and its implications for dignity in mobile COVID-19 testing. SAGE Open Nurs. 2021;7:23779608211029096. doi: 10.1177/23779608211029096.
26. Vinjerui KH, Elgersma IH, Fretheim A. Increased COVID-19 testing rates following combined door-to-door and mobile testing facility campaigns in Oslo, Norway, a difference-in-difference analysis. Int J Environ Res Public Health. 2021;18(21). doi: 10.3390/ijerph182111078.
27. Kost GJ, Ferguson WJ, Kost LE. Principles of point of care culture, the spatial care path, and enabling community and global resilience. J Intl Fed Clin Chem Lab Med. 2014;25:134-153.
28. Kost GJ, Ferguson WJ, Hoe J, Truong AT, Banpavichit A, Kongpila S. The Ebola Spatial Care Path™: Accelerating point-of-care diagnosis, decision making, and community resilience in outbreaks. Am J Disaster Med. 2015;10(2):121-43.
29. Kost GJ, Ferguson WJ, Truong A-T, Prom D, Hoe J, Banpavichit A, et al. The Ebola Spatial Care Path: Point-of-care lessons learned for stopping outbreaks. Clin. Lab. Int. 2015;39:6–14.
30. Kost GJ, Ferguson WJ. Spatial care paths strengthen links in the chain of global resilience: Disaster caches, prediabetes, Ebola Virus Disease, and the future of point of care. Point of Care. 2016;15:43-58.
31. Kost GJ, Pratumvinit B. Diabetes spatial care paths, leading edge HbA1c testing, facilitation thresholds, proactive-preemptive strategic intelligence, and unmanned aerial vehicles in limited-resource countries. Point of Care. 2017;16:12-31.
32. Kost GJ, Zadran A, Duong TTB, Pham TT, Ho AVD, Nguyen NV, et al. Point-of-care diagnosis of acute myocardial infarction in Central Vietnam: International exchange, needs assessment, and spatial care paths. Point of Care. 2018;17(3):73-92.
33. Ferguson WJ, Louie RF, Tang CS, Vy JH, Wallace AP, Peng LS, et al. Geographic information systems can enhance crisis standards of care during complex emergencies and disasters. Point of Care. 2012;11:184-190.
34. Ferguson WJ, Louie RF, Katip P, Kost GJ. Use of geographic information systems for placement and management of point-of-care technologies in small-world networks. In Kost GJ, Ed., Curtis CM, Assoc. Ed. Global Point of Care: Strategies for Disasters, Emergencies, and Public Health Resilience. Washington DC: AACC Press-Elsevier; 2015**.** Chapter 35, p. 393-404.
35. Ferguson WJ, Kemp K, Kost G. Using a geographic information system to enhance patient access to point-of-care diagnostics in a limited-resource setting. Int J Health Geogr. 2016;15:10. doi: 10.1186/s12942-016-0037-9.
36. Kost GJ. Geospatial science and point-of-care testing: Creating solutions for population access, emergencies, outbreaks, and disasters. Front Public Health. 2019;7(329):1-31.
37. Castro-Portillo E, López-Izquierdo R, Sanz-García A, Ortega GJ, Delgado-Benito JF, Castro Villamor MA, et al. Role of prehospital point-of-care N-terminal pro-brain natriuretic peptide in acute life-threatening cardiovascular disease. Int J Cardiol. 2022;S0167-5273(22):923-928. doi: 10.1016/j.ijcard.2022.06.025.
38. Jones C, Lightowler B. The efficacy of the HEART score in prehospital settings. J Paramed Pract. 2022;14(5):198-211.
39. Pavlovsky T, Obadia M, Ragot S, Douay B, Casalino E, Ghazali DA. Predictors of risk stratification and value of point-of-care of high-sensitivity cardiac troponin-I in EMS management of non-ST-segment elevation myocardial infarction: A retrospective study. Prehosp Disaster Med. 2022;37(3):365-372.
40. Cooper JG, Ferguson J, Donaldson LA, Black KMM, Horrill JL, Davidson EM, et al. Could high-sensitivity cardiac troponin testing rule out acute myocardial infarction in the prehospital setting? J Am Coll Cardiol. 2021;78(23):2392-2394.
41. Cooper JG, Ferguson J, Donaldson LA, Black KMM, Livock KJ, Horrill JL, et al. The ambulance cardiac chest pain evaluation in Scotland study (ACCESS): A prospective cohort study. Ann Emerg Med. 2021;77(6):575-588.
42. Koper LH, Frenk LDS, Meeder JG, van Osch FHM, Bruinen AL, Janssen MJW, et al. URGENT 1.5: diagnostic accuracy of the modified HEART score, with fingerstick point-of-care troponin testing, in ruling out acute coronary syndrome. Neth Heart J. 2022;30(7-8):360-369.
43. Lee TH, Chen LC, Wang E, Wang CC, Lin YR, Chen WL. Development of an electrochemical immunosensor for detection of cardiac troponin I at the point-of-care. Biosensors (Basel). 2021;11(210):1-9.
44. Martín-Rodríguez F, Sanz-García A, Castro-Portillo E, Delgado-Benito JF, Del Pozo Vegas C, Ortega Rabbione G, et al. Prehospital troponin as a predictor of early clinical deterioration. Eur J Clin Invest. 2021;51(11):e13591.
45. Stopyra JP, Snavely AC, Smith LM, Harris RD, Nelson RD, Winslow JE, et al. Prehospital use of a modified HEART pathway and point-of-care troponin to predict cardiovascular events. PLoS ONE. 2020;15(10): e0239460.
46. Stopyra JP, Snavely AC, Scheidler JF, Smith LM, Nelson RD, Winslow JE, et al. Point-of-care troponin testing during ambulance transport to detect acute myocardial infarction. Prehosp Emerg Care. 2020;24(6):751-759.
47. Alghamdi A, Alotaibi A, Alharbi M, Reynard C, Body R. Diagnostic performance of prehospital point-of-care troponin tests to rule out acute myocardial infarction: A systematic review. Prehosp Disaster Med. 2020;35(5):567-573.
48. Alghmadi A, Body R. BET 1: Prehospital cardiac troponin testing to 'rule out' acute coronary syndromes using point of care assays. Emerg Med J. 2018;35(9):572-574.
49. Harjola P, Miró Ò, Martín-Sánchez FJ, Escalada X, Freund Y, Penaloza A, et al. Pre-hospital management protocols and perceived difficulty in diagnosing acute heart failure. ESC Heart Fail. 2020;7(1):289-296.
50. Johannessen TR, Vallersnes OM, Halvorsen S, Larstorp ACK, Mdala I, Atar D. Pre-hospital one-hour troponin in a low-prevalence population of acute coronary syndrome: OUT-ACS study. Open Heart. 2020;7:e001296.
51. Kaier TE, Stengaard C, Marjot J, Sørensen JT, Alaour B, Stavropoulou-Tatla S, et al. Cardiac myosin-binding protein C to diagnose acute myocardial infarction in the pre-hospital setting. J Am Heart Assoc. 2019;8(15):e013152. doi: 10.1161/jaha.119.013152.
52. Alghamdi A, Body R. BET 1: Prehospital cardiac troponin testing to 'rule out' acute coronary syndromes using point of care assays. Emerg Med J. 2018;35(9):572-574.
53. Rasmussen MB, Stengaard C, Sørensen JT, Riddervold IS, Hansen TM, Giebner M, et al. Predictive value of routine point-of-care cardiac troponin T measurement for prehospital diagnosis and risk-stratification in patients with suspected acute myocardial infarction. Eur Heart J Acute Cardiovasc Care. 2019;8(4):299-308.
54. Stengaard C, Sørensen JT, Ladefoged SA, Lassen JF, Rasmussen MB, Pedersen CK, et al. The potential of optimizing prehospital triage of patients with suspected acute myocardial infarction using high-sensitivity cardiac troponin T and copeptin. Biomarkers. 2017;22(3-4):351-360.
55. Ezekowitz JA, Welsh RC, Weiss D, Chan M, Keeble W, Khadour F, et al. Providing rapid out of hospital acute cardiovascular treatment 4 (PROACT-4). J Am Heart Assoc. 2015;4(12):1-9.
56. Tideman PA, Tirimacco R, Senior DP, Setchell JJ, Huynh LT, Tavella R. Impact of a regionalised clinical cardiac support network on mortality among rural patients with myocardial infarction. Med J Aust. 2014;200(3):157-160.
57. Stengaard C, Thorsted Sørensen J, Terkelsen CJ. Prehospital point of care testing of biomarkers has diagnostic value in relation to acute myocardial infarction. Ugeskr Laeger. 2013;175(4):186-189.
58. Venturini JM, Stake CE, Cichon ME. Prehospital point-of-care testing for troponin: are the results reliable? Prehosp Emerg Care. 2013;17(1):88-91.
59. Prosen G, Klemen P, Štrnad M, Grmec S. Combination of lung ultrasound (a comet-tail sign) and N-terminal pro-brain natriuretic peptide in differentiating acute heart failure from chronic obstructive pulmonary disease and asthma as cause of acute dyspnea in prehospital emergency setting. Crit Care. 2011;15(2):R114.
60. Sørensen JT, Terkelsen CJ, Steengaard C, Lassen JF, Trautner S, Christensen EF, et al. Prehospital troponin T testing in the diagnosis and triage of patients with suspected acute myocardial infarction. Am J Cardiol. 2011;107(10):1436-1440.
61. Kost GJ, Kost LE, Suwanyangyuen A, Cheema SK, Curtis C, Sumner S, et al. Emergency cardiac biomarkers and point-of-care testing: Optimizing acute coronary syndrome care using small-world networks in rural settings. Point of Care. 2010;9:53-64.
62. Di Serio F, Lovero R, Leone M, De Sario R, Ruggieri V, Varraso L, et al. Integration between the tele-cardiology unit and the central laboratory: methodological and clinical evaluation of point-of-care testing cardiac marker in the ambulance. Clin Chem Lab Med. 2006;44(6):768-773.
63. Owens CG, McClelland AJ, Walsh SJ, Smith BA, Tomlin A, Riddell JW, et al. Prehospital 80-LAD mapping: does it add significantly to the diagnosis of acute coronary syndromes? J Electrocardiol. 2004;37 Suppl:223-232.
64. Schuchert A, Hamm C, Scholz J, Klimmeck S, Goldmann B, Meinertz T. Prehospital testing for troponin T in patients with suspected acute myocardial infarction. Am Heart J. 1999;138(1 Pt 1):45-48.
65. Matthes A, Wolf F, Wilde E, Bleidorn J, Markwart R. Point-of-care measurement of C-reactive protein promotes de-escalation of treatment decisions and strengthens the perceived clinical confidence of physicians in out-of-hours outpatient emergency medical services. BMJ Open. 2023;13(5):e069453. doi: 10.1136/bmjopen-2022-069453.
66. Collopy KT, Westmoreland A, Powers WF. Patient care alterations after point-of-care laboratory testing during critical care transport. Air Med J. 2022;41(4):370-375.
67. Morton S, Avery P, Payne J, OMeara M. Arterial blood gases and arterial lines in the prehospital setting: a systematic literature review and survey of current United Kingdom Helicopter Emergency Medical Services. Air Med J. 2022;41:201-208.
68. Murali A, Guyette FX, Martin-Gill C, Jones M, Kravetsky M, Wheeler SE. Implementation and challenges of portable blood gas measurements in air medical transport. Clin Chem Lab Med. 2022;60(6):859-866.
69. Cini S, Urbanelli A, Montemerani S, Ramacciani Isemann C, Righi L. Point-of-care blood gas analysis as a tool for COVID-19 patients: a prehospital setting experience. Recenti Prog Med. 2021;112(12):821-823.
70. Gruebl T, Ploeger B, Wranze-Bielefeld E, Mueller M, Schmidbauer W, Kill C, et al. Point-of-care testing in out-of-hospital cardiac arrest: a retrospective analysis of relevance and consequences. Scand J Trauma Resusc Emerg Med. 2021;29(128):1-10.
71. Nawrocki J, Furian M, Buergin A, Mayer L, Schneider S, Mademilov M, et al. Validation of a portable blood gas analyzer for use in challenging field conditions at high altitude. Front Physiol. 2021;11:600551. doi: 10.3389/fphys.2020.600551.
72. McPherson MD. Point-of-care blood tests in decision-making for people over 65 with acute frailty. J Paramed Pract. 2019;11(3):106-115.
73. Shin J, Lim YS, Kim K, Lee HJ, Lee SJ, Jung E, et al. Initial blood pH during cardiopulmonary resuscitation in out-of-hospital cardiac arrest patients: a multicenter observational registry-based study. Crit Care. 2017;21(1):322.
74. Mikkelsen S, Wolsin-Hansen J, Nybo M, Maegaard CU, Jepsen S. Implementation of the ABL-90 blood gas analyzer in a ground-based mobile emergency care unit. SJTREM. 2015;23:54 (letter). doi: 10.1186/s13049-015-0134-y
75. Di Serio F, Petronelli MA, Sammartino E. Laboratory testing during critical care transport: point-of-care testing in air ambulances. Clin Chem Lab Med. 2010;48(7):955-961.
76. Jousi M, Reitala J, Lund V, Katila A, Leppäniemi A. The role of pre-hospital blood gas analysis in trauma resuscitation. World J Emerg Surg. 2010;5(10):1-7.
77. Vos G, Engel M, Ramsay G, van Waardenburg D. Point-of-care blood analyzer during the interhospital transport of critically ill children. Europ J Emerg Med. 2006;13:304-307.
78. Gruszecki AC, Hortin G, Lam J, Kahler D, Smith D, Vines J, et al. Utilization, reliability, and clinical impact of point-of-care testing during critical care transport: six years of experience. Clin Chem. 2003;49(6):1017-1019.
79. Backer HD, Collins S. Use of a handheld, battery-operated chemistry analyzer for evaluation of heat-related symptoms in the backcountry of Grand Canyon National Park: a brief report. Ann Emerg Med. 1999;33(4):418-422.
80. Herr DM, Newton NC, Santrach PJ, Hankins DG, Burritt MF. Airborne and rescue point-of-care testing. Am J Clin Pathol. 1995;104(4 Suppl 1, Kost Ed.):S54-S58.
81. Hill J, Gothard DM, McLean MM. Prehospital blood glucose testing as a predictor of impending hypotension in adult trauma patients. Air Med J. 2020;39(1):20-23.
82. Remick K, Redgate C, Ostermayer D, Kaji AH, Gausche-Hill M. Prehospital glucose testing for children with seizures: A proposed change in management. Prehosp Emerg Care. 2017;21(2):216-221.
83. Lerner EB, Billittier IV AJ, Lance DR, Janicke DM, Teuscher JA. Can paramedics safely treat and discharge hypoglycemic patients in the field? Am J Emerg Med. 2003;21(2):115-120.
84. Novak A, Cherry J, Ali N, Smith I, Bowen J, Ray J, et al. Point-of-care blood testing with secondary care decision support for frail patients. J Paramed Pract. 2022;14(2):54-62
85. Galvagno SM, Sikorski RA, Floccare DJ, Rock P, Mazzeffi MA, DuBose JJ, et al. Prehospital point of care testing for the early detection of shock and prediction of lifesaving interventions. Shock. 2020;54(6):710-716.
86. Martín-Rodríguez F, López-Izquierdo R, Medina-Lozano E, Ortega Rabbione G, Del Pozo Vegas C, Carbajosa Rodríguez V, et al. Accuracy of prehospital point-of-care lactate in early in-hospital mortality. Eur J Clin Invest. 2020;50(12):e13341. doi: 10.1111/eci.13341.
87. Martín-Rodríguez F, López-Izquierdo R, Castro Villamor MA, Mangas IM, Del Brío Ibáñez P, Delgado Benito JF, et al. Prognostic value of lactate in prehospital care as a predictor of early mortality. Am J Emerg Med. 2019;37(9):1627-1632.
88. Krass I, Carter R, Mitchell B, Mohebbi M, Shih STF, Trinder P, et al. Pharmacy diabetes screening trial (PDST): Outcomes of a national clustered RCT comparing three screening methods for undiagnosed type 2 diabetes (T2DM) in community pharmacy. Diab Res Clin Pract. 2023;197:110566. doi: 10.1016/j.diabres.2023.110566.
89. Ventura IJ, Zadran A, Ho AVD, Zadran L, Thuan DTB, Pham TT, et al. Rapid diagnosis and effective monitoring of diabetes mellitus in central Vietnam: Point-of-care needs, improved patient access, and spatial care paths for enhanced public health. Point of Care. 2019;18:1-8.
90. Shephard M, Shephard A, McAteer B, Regnier T, Barancek K. Results from 15 years of quality surveillance for a National Indigenous Point-of-Care Testing Program for diabetes. Clin Biochem. 2017;50(18):1159-1163.
91. Motta LA, Shephard MDS, Brink J, Lawson S, Rheeder P. Point-of-care testing improves diabetes management in a primary care clinic in South Africa. Prim Care Diabetes. 2017;11(3):248-253.
92. Kost GJ, Kanoksilp A, Mecozzi DM, Sonu R, Curtis C, Yu JN. Point-of-need hemoglobin A1c for evidence-based diabetes care in rural small-world networks: Khumuang Community Hospital, Buriram, Thailand. Point of Care. 2011;10:28-33.
93. Spaeth BA, Shephard MD, Schatz S. Point-of-care testing for haemoglobin A1c in remote Australian Indigenous communities improves timeliness of diabetes care. Rural Remote Health. 2014;14(4):2849.
94. Martin DD, Shephard MD, Freeman H, Bulsara MK, Jones TW, Davis EA, et al. Point-of-care testing of HbA1c and blood glucose in a remote Aboriginal Australian community. Med J Aust. 2005;182(10):524-527.
95. Shephard MD, Mazzachi BC, Shephard AK, McLaughlin KJ, Denner B, Barnes G. The impact of point of care testing on diabetes services along Victoria's Mallee Track: results of a community-based diabetes risk assessment and management program. Rural Remote Health. 2005;5(3):371.
96. Schober P, Bossers SM, Koolwijk J, Terra M, Schwarte LA. Prehospital coagulation measurement by a portable blood analyzer in a helicopter emergency medical service (HEMS). Am J Emerg Med. 2021;46:137-140.
97. Beynon C, Erk AG, Potzy A, Mohr S, Popp E. Point of care coagulometry in prehospital emergency care: an observational study. Scand J Trauma Resusc Emerg Med. 2015;23:58.
98. Rumpf TH, Krizmaric M, Grmec S. Capnometry in suspected pulmonary embolism with positive D-dimer in the field. Crit Care. 2009;13(6):R196
99. Christensen HM, Pietersen PI, Laursen CB, Wittrock D, Nadim G, Jørgensen G, et al. Patients' perspectives on point-of-care diagnostics and treatment by emergency medical technicians in acute COPD exacerbations: A qualitative study. Scand J Trauma Resusc Emerg Med. 2022;30(1):11.
100. Ienghong K, Cheung LW, Tiamkao S, Bhudhisawasdi V, Apiratwarakul K. The utilization of handheld ultrasound devices in a prehospital setting. Prehosp Disaster Med. 2022;37(3):355-359. doi: 10.1017/s1049023x22000644.
101. Dubecq C, Dubourg O, Morand G, Montagnon R, Travers S, Mahe P. Point-of-care ultrasound for treatment and triage in austere military environments. J Trauma Acute Care Surg. 2021;91(2S Suppl 2):S124-129.
102. Nadim G, Laursen CB, Pietersen PI, Wittrock D, Sørensen MK, Nielsen LB, et al. Prehospital emergency medical technicians can perform ultrasonography and blood analysis in prehospital evaluation of patients with chronic obstructive pulmonary disease: a feasibility study. BMC Health Serv Res. 2021;21(1):290.
103. Schoeneck JH, Coughlin RF, Baloescu C, Cone DC, Liu RB, Kalam S, et al. Paramedic-performed prehospital point-of-care ultrasound for patients with undifferentiated dyspnea: A pilot study. West J Emerg Med. 2021;22(3):750-755.
104. Sabatino V, Caramia MR, Curatola A, Vassallo F, Deidda A, Cinicola B, et al. Point-of-care ultrasound (POCUS) in a remote area of Sierra Leone: impact on patient management and training program for community health officers. J Ultrasound. 2020;23(4):521-527.
105. Bobbia X, Abou-Badra M, Hansel N, Pes P, Petrovic T, Claret PG, et al. Changes in the availability of bedside ultrasound practice in emergency rooms and prehospital settings in France. Anaesth Crit Care Pain Med. 2018;37(3):201-205.
106. Chin EJ, Chan CH, Mortazavi R, Anderson CL, Kahn CA, Summers S, et al. A pilot study examining the viability of a prehospital assessment with ultrasound for emergencies (PAUSE) protocol. J Emerg Med. 2013;44(1):142-149.
107. Advance Health Services Cooperative. Mobile Diagnostic Clinic. Philippine Cooperative Central Fund Federation Bldg, 20 Gov. M. Roa St, Cebu City, 6000 Cebu.
108. Söderqvist M, Virta J, Kämäräinen A. Substance abuse among emergency medical services patients. A pilot study on the clinical impact of an on-site oral fluid screening test. Point of Care. 2018;17(2):47-49.
109. Levens AD, den Haan MC, Jukema JW, Heringa M, van den Hout WB, Moes D, et al. Feasibility of community pharmacist-initiated and point-of-Care CYP2C19 genotype-guided de-escalation of oral P2Y12 inhibitors. Genes. 2023;14(3).
110. Do NTT, Vu TVD, Greer RC, Dittrich S, Vandendorpe M, Pham NT, et al. Implementation of point-of-care testing of C-reactive protein concentrations to improve antibiotic targeting in respiratory illness in Vietnamese primary care: a pragmatic cluster-randomised controlled trial. Lancet Infect Dis. 2023. doi: 10.1016/s1473-3099(23)00125-1.
111. Mital S, Kelly D, Hughes C, Nosyk B, Thavorn K, Nguyen HV. Estimated cost-effectiveness of point-of-care testing in community pharmacies vs. self-testing and standard laboratory testing for HIV. AIDS (London, England). 2023;37(7):1125-35.
112. Sheehan Y, Cunningham EB, Cochrane A, Byrne M, Brown T, McGrath C, et al. A 'one-stop-shop' point-of-care hepatitis C RNA testing intervention to enhance treatment uptake in a reception prison: the PIVOT study. J Hepatology. 2023. doi: 10.1016/j.jhep.2023.04.019.
113. Department of Health, Philippines. USAID to expedite patients’ access to TB care. <https://www.pna.gov.ph/articles/1174445> Accessed November 22, 2023.
114. Beeman A, Gonzalez Marques C, Tang OY, Uwamahoro C, Jarmale S, Mutabazi Z, et al. Factors associated with HIV testing among patients seeking emergent injury care in Kigali, Rwanda. Afr J Emerg Med. (2022) 12(3):281-286.
115. Zadran A, Ho AVD, Zadran L, Ventura-Curiel IJ, Pham T-T, Kost GJ. Optimizing public health preparedness for highly infectious diseases in Central Vietnam. Diagnostics. (2022) 12(2047):1-29. doi.org/10.3390/diagnostics12092047
116. World Health Organization. Consolidated Guidelines on HIV Prevention, Testing, Treatment, Service Delivery and Monitoring: Recommendations for a Public Health Approach. Geneva: WHO. (2021), 592 pages. <https://www.who.int/publications/i/item/9789240031593> Accessed November 22, 2023.
117. Vallely AJ, Pomat WS, Homer C, Guy R, Luchters S, Mola GDL, et al. Point-of-care testing and treatment of sexually transmitted infections to improve birth outcomes in high-burden, low-income settings: Study protocol for a cluster randomized crossover trial (the WANTAIM Trial, Papua New Guinea). Welcome Open Res. (2019) 4:53. doi: 10.12688/wellcomeopenres.15173.2.
118. West K. Rapid HIV testing: a new standard of care to protect prehospital providers. J Emer Med Serv. (2007) 32(5):40-41. doi: 10.1016/S0197-2510(07)72192-7.
119. West K. HIV: test results in minutes: reviewing the prehospital occupational risk of acquiring HIV & the availability & value of rapid post-exposure testing. J Emer Med Serv. (2004) 29(7):68-73.
120. Metelmann C, Metelmann B, Scheer C, Gründling M, Henkel B, Hahnenkamp K, et al. Sepsis detection in emergency medicine: Results of an interprofessional survey on sepsis detection in prehospital emergency medicine and emergency departments. Anaesthesist. (2018) 67(8):584-591.
121. Donoso-Calero MI, Martín Conty JL, López-Izquierdo R, Sanz-García A, Dileone M, Polonio-López B, et al. Prehospital seizures: Short-term outcomes and risk stratification based in point-of-care testing. European J Clin Invest. 2023:e14042.
122. Ebinger M, Siegerink B, Kunz A, Wendt M, Weber JE, Schwabauer E, et al. Association between dispatch of mobile stroke units and functional outcomes among patients with acute ischemic stroke in Berlin. JAMA. 2021;325(5):454-466.
123. Wendt M, Ebinger M, Kunz A, Rozanski M, Waldschmidt C, Weber JE, et al. Improved prehospital triage of patients with stroke in a specialized stroke ambulance. Results of the pre-hospital acute neurological therapy and optimization of medical care in stroke study. Stroke 2015;46:740-745.
124. Ebinger M, Winter B, Wendt M, Weber JE, Waldschmidt C, Rozanksi M, et al. Effect of the use of ambulance-based thrombolysis on time to thrombolysis in acute ischemic stroke. JAMA 2014;311(16):1622-1631.
125. Walter S, Kostopoulos P, Haas A, Keller I, Lesmeister M, Schlechtriemen T, et al. Diagnosis and treatment of patients with stroke in a mobile stroke unit versus in hospital: a randomized controlled trial. Lancet Neurol. 2012;11:397-404.
126. Chan J, Griffith LE, Costa AP, Leyenaar MS, Agarwal G. Community paramedicine: a systematic review of program descriptions and training. Canadian J Emer Med. 2019;21:749-761.
127. Van Vuuren J, Thomas B, Agarwal G, MacDermott S, Kinsman L, O’Meara P, et al. Reshaping healthcare delivery for elderly patients: the role of community paramedicine; a systematic review. BMC Health Services Research. 2021;21:29.
128. Thirumalai M, Zengul AG, Evans E. Challenges and lessons learned from a telehealth community paramedicine program for the prevention of hypoglycemia: pre-post pilot feasibility study. JMIR Diabetes. 2021;6:e26941.
129. Rural Health Information Hub. Community Paramedicine. <https://www.ruralhealthinfo.org/topics/community-paramedicine> Accessed November 22, 2023.
130. Paramedic Real-world Example 1. <https://www.albertahealthservices.ca/ems/Page16487.aspx> Accessed November 22, 2023.
131. Paramedic Real-world Example 2. <http://www.bcehs.ca/our-services/programs-services/community-paramedicine> Accessed November 22, 2023.
132. Leff B, Burton L, Guido S., Greenough WB, Steinwachs D, Burton JR. Home hospital program: a pilot study. J Am Geriatr Soc. 1999;47:697-702.
133. Leff B, Burton L, Mader SL, Naughton B, Burl J, Inouye SK, et al. Hospital at home: feasibility and outcomes of a program to provide hospital-level care at home for acutely ill older patients. Ann Intern Med. (2005) 143:798-808.
134. Leong MQ, Lim CW, Lai YF. Comparison of hospital-at-home models: a systematic review of reviews. BMJ Open. 2021;11:e043285.
135. Balatbat C, Kadakia KT, Dzau VJ, Offodile AC. No place like home: Hospital at home as a post-pandemic frontier for care delivery innovation. NEJM Catalyst. 2021. <https://catalyst.nejm.org/doi/pdf/10.1056/CAT.21.0237> Accessed November 22, 2023.
136. Lippert M, Semmens S, Tacey L, Rent T, Defoe K, Bucsis M, et al. The Hospital at home program: no place like home. Curr Oncol. 2017;24:23-27.
137. Johns Hopkins Hospital at Home online resource: <https://www.hospitalathome.org/> Accessed November 22, 2023.
138. Home Hospital Real-world Example 1: <https://www.islandhealth.ca/our-services/hospital-home-services/hospital-home> Accessed November 22, 2023.
139. Home Hospital Real-world Example 2: <https://www.ualberta.ca/folio/2020/02/virtual-hospital-cuts-ems-calls-er-visits-and-hospital-admissions-for-high-use-patients.html> Accessed November 22, 2023.
140. Flemons K, Baylis B, Khan AZ, Kirkpatrick AW, Whitehead K, Moeini S. The use of drones for the delivery of diagnostic test kits and medical supplies to remote First Nations communities during Covid-19. Am J Infect Control. 2022;50(8):849-856.
141. Katariya M, Chung DCK, Minife T, Gupta H, Zahidi AAA, Liew OW, et al. Drone inflight mixing of biochemical samples. Anal Biochem. 2018;545:1-3.
142. Amukele TK, Hernandez J, Snozek CLH, Wyatt RG, Douglas M, Amini R, et al. Drone transport of chemistry and hematology samples over long distances. Am J Clin Pathol. 2017;148(5):427-435.
143. Amukele T, Ness PM, Tobian AA, Boyd J, Street J. Drone transportation of blood products. Transfusion. 2017;57(3):582-588.
144. Amukele TK, Street J, Carroll K, Miller H, Zhang SX. Drone transport of microbes in blood and sputum laboratory specimens. J Clin Microbiol. 2016;54(10):2622-2625.
145. Claesson A, Fredman D, Svensson L, Ringh M, Hollenberg J, Nordberg P, et al. Unmanned aerial vehicles (drones) in out-of-hospital-cardiac-arrest. Scand J Trauma Resusc Emerg Med. 2016;24(1):124.
146. Priye A, Wong S, Bi Y, Carpio M, Chang J, Coen M, et al. Lab-on-a-drone: Toward pinpoint deployment of smartphone-enabled nucleic acid-based diagnostics for mobile health care. Anal Chem. 2016;88(9):4651-4660.
147. Amukele TK, Sokoll LJ, Pepper D, Howard DP, Street J. Can unmanned aerial systems (drones) be used for the routine transport of chemistry, hematology, and coagulation laboratory specimens? PLoS One. 2015;10(7):e0134020. doi: 10.1371/journal.pone.0134020

Compendium Version 4.2 • November 22, 2023
