# Supplementary material for: Using geographic rescue time contours, point-of-care strategies, and spatial care paths to prepare island communities for global warming, rising oceans, and weather disasters
Source: Int J Health Geogr. 2023 Dec 20;22:38. doi: 10.1186/s12942-023-00359-y (PMC10731708; doi:10.1186/s12942-023-00359-y)
Supplement: Supplementary file 2 — Additional file 2. Collection Tool for Needs Assessment Data. [file 12942_2023_359_MOESM2_ESM.docx]

**Collection Tool for Needs Assessment Data**

**Title: Using Geographic Time Contours, Point-of-care Strategies, and Spatial Care Paths to Prepare Island Communities for Global Warming, Rising Oceans, and Weather Disasters**

**Goal: To design point-of-care testing and geospatial care paths that improve outcomes.**

**Definition: Point-of-care testing is medical testing at or near the site of patient care.**

**Table of contents Page**

Section I. Facility Overview and Demographics………………………….……1

Section II. Point-of-Care Testing (POCT) ……………………………………..3

Section III. Patient Transport and Critical Care Access……………………… 7

Section IV. Clinical Laboratory…………………………………………..……15

Section V. Medical Problem Solving in the Community……….…...…………20

Section VI. Summary of Needs Assessment Observations…………………….21

Accession Number __________________

GPS Coordinates: _____ (latitude) _____ (longitude)

Investigators ____________________ (Team members contributing these data)

**SECTON I. FACILITY OVERVIEW AND DEMOGRAPHICS**

1.1 Type of facility ( ) Community hospital ( ) Regional hospital ( ) University

( ) Private hospital ( ) Military hospital ( ) Emergency room

( ) Drive-thru, walk-up ( ) Urgent care ( ) Other…………

1.2 Size of hospital or facility ฀ ( ) Small ฀ ( ) Medium ฀ ( ) Large

1.3 Number of beds

1.4 Number of physicians

1.5 Number of pharmacists

1.6 Number of nurses

1.7 Number of lab personnel: Medical technologist Medical science … Phlebotomists…

1.8 Number of ambulances

1.9 Is there an emergency room (ER)?

฀ ( ) Yes ( ) No ฀ ( ) Don’t know

1.10 Are there operating rooms (ORs)?

฀ ( ) Yes, identify number of ORs ( ) No ฀ ( ) Don’t know

1.11 Are there intensive care units (ICUs)?

฀ ( ) Yes ( ) No ฀ ( ) Don’t know

- If so, identify number of ICUs rooms

1.12 Are there neonatal intensive care units (NICUs)?

฀ ( ) Yes ( ) No

- If so, identify number of NICUs rooms

1.13 Are there cardiac care units (CCUs)?

฀ ( ) Yes ( ) No

- If so, identify number of CCU beds

1.14 Is there a labor room (LR)?

- ( ) Yes ( ) No

1.15 Has the hospital been certified by ISO, The Joint Commission (TJC), Government, or other accreditation agency(ies), such as the College of American Pathologists (CAP)?

฀ ( ) ISO ฀ ( ) TJC ( ) CAP ( ) Philippine Government, specify ______________

( ) Other , specify

1.16 What percentages of patients are: ___ acute ___ chronic

1.17 How many patients visit your facility each day on average? ___

**SECTION II. POINT-OF-CARE TESTING (POCT)**

2.1 Is there a point-of-care (POC) testing or bedside testing program?

฀ ( ) Yes ฀ ( ) No ฀

2.2 Is there a POC testing director responsible for quality and accuracy?

฀ ( ) Yes ฀ ( ) No ฀

2.3 Is there a POC Coordinator who manages and oversees POC testing on a daily basis?

( ) Yes ฀ ( ) No

2.4 List the tests performed at the patient bedside or near the patient in the following departments and who performs the testing:

Ward (a large room in a hospital where a number of patients often needing similar treatment are cared for) Who?

OR Who?

ER Who?

LR Who?

ICU Who?

NICU Who?

CCU Who?

Sites outside the hospital (specify type)

Who is responsible for POC testing outside the hospital?

2.5 Specify the POC tests or devices that your hospital does NOT have, but would like to perform or use in the following departments in the future:

Ward

OR

ER

LR

ICU

NICU

CCU

Sites outside the hospital (specify type)

Other

2.6. Please rank the POC tests that you need the most and where the diagnostic tests should be located (e.g., patient home, primary care, clinic, health promoting hospital, emergency room, labor and delivery, OR, ICU, NICU, CCU, ward, or ambulance).

**Diagnostic tests Rank Where to perform**

Blood typing (A, B, O, Rh) _____ _____________

Rapid microbiology tests (e.g., COVID-19) _____ _____________

Coagulation (PT/ INR) _____ _____________

Blood gases (pH, pO_2_, pCO_2_) _____ _____________

Chemistry/ electrolytes/ ionized calcium _____ _____________

Hematology (CBC, differential, platelet) _____ _____________

Cardiac biomarkers (cTnT, cTnI, others) _____ _____________

Pulse oximeter (O_2_ saturation) _____ _____________

2.7 For POC testing, rank the top five diagnostic tests from most useful (1) to least useful (5):

1. (most useful) _____________ 2. _____________ 3. _____________

4. _____________ 5. _____________

2.8 Specify and rank POC tests/ devices used most frequently in your **ER.**

1. _____________ 6. _____________

2. _____________ 7. _____________

3. _____________ 8. _____________

4. _____________ 9. _____________

5. _____________ 10. _____________

2.9 Specify and rank POC tests/ devices used most frequently in your **ICU.**

1. _____________ 6. _____________

2. _____________ 7. _____________

3. _____________ 8. _____________

4. _____________ 9. _____________

5. _____________ 10. _____________

2.10 Specify and rank POC tests/ devices used most frequently in your **OR.**

1. _____________ 6. _____________

2. _____________ 7. _____________

3. _____________ 8. _____________

4. _____________ 9. _____________

5. _____________ 10. _____________

2.11 Specify and rank POC tests/ devices used most frequently in your **NICU.**

1. _____________ 6. _____________

2. _____________ 7. _____________

3. _____________ 8. _____________

4. _____________ 9. _____________

5. _____________ 10. _____________

2.12 Specify and rank POC tests/ devices used most frequently in your **CCU.**

1. _____________ 6. _____________

2. _____________ 7. _____________

3. _____________ 8. _____________

4. _____________ 9. _____________

5. _____________ 10. _____________

2.13 Specify and rank POC tests/ devices used most frequently in your **LR.**

1. _____________ 6. _____________

2. _____________ 7. _____________

3. _____________ 8. _____________

4. _____________ 9. _____________

5. _____________ 10. _____________

2.14 Specify and rank POC tests/ devices used most frequently in your **ward.**

1. _____________ 6. _____________

2. _____________ 7. _____________

3. _____________ 8. _____________

4. _____________ 9. _____________

5. _____________ 10. _____________

2.15 Specify and rank POC tests that are frequently used in your **ambulances.**

1. _____________ 6. _____________

2. _____________ 7. _____________

3. _____________ 8. _____________

4. _____________ 9. _____________

5. _____________ 10. _____________

2.16 Which POC tests would you like to place in local primary care centers, clinics, health promoting hospitals, or POC sites to improve patient access to diagnosis? List and rank:

1. _____________ 6. _____________

2. _____________ 7. _____________

3. _____________ 8. _____________

4. _____________ 9. _____________

5. _____________ 10. ____________

2.17 Which POC tests would you place in patient homes, so they can do their own monitoring and call in, email, or directly transmit via Internet results to you or a physician? List and rank:

1. _____________ 6. _____________

2. _____________ 7. _____________

3. _____________ 8. _____________

4. _____________ 9. _____________

5. _____________ 10. ____________

2.18 Suppose a patient is suddenly critically ill at home. Which POC tests should ambulance staff (e.g., paramedics) perform while transporting the patient to speed up diagnosis and appropriate treatment. List and rank:

1. _____________ 6. _____________

2. _____________ 7. _____________

3. _____________ 8. _____________

4. _____________ 9. _____________

5. _____________ 10. ____________

2.19 Who mainly performs the POC testing?

( ) Physician ฀ ฀ ( ) Nurse ฀( ) Lab staff ฀ ฀ ( ) Other

2.20 Do patients themselves perform testing or monitoring at their sites of patient care?

( ) Yes ฀ ( ) No

If so, which ones (list)……………………………………….

2.21 After they leave the hospital, do patients perform testing at their homes?

( ) Yes ฀ ( ) No

If so, what is that test? Please specify…………………

2.22 Do patients consult physicians about the test results produced by themselves?

( ) Yes ฀ ( ) No

2.23 Do patients or their families receive training on how to perform testing themselves?

฀ ( ) Yes ฀ ( ) No

2.24 What types of additional POC tests should be provided to your hospital?

Please specify………………………………………………………………………………

2.25 What types of additional tests should be provided for patients to perform themselves?

Please specify………………………………………………………………………………

2.26 To facilitate patient access to healthcare delivery, where should POC resources be placed in your health network?

( ) In patient homes, allowing them to perform self-testing (e.g., rapid antigen self-testing)

( ) In a primary care site or in a health promoting hospital close to patient homes and work

( ) In a hospital close to where patients live, and if so, how close _______ (km)

( ) In a regional hospital, and if so, at what maximum distance from homes ____ (km)

( ) In drive-throughs, walk-ins, pharmacies, or other sites providing COVID-19 testing

( ) Other, specify……………………………………………..

**SECTION III. PATIENT TRANSPORT AND CRITICAL CARE ACCESS ACCESS**

- **Ambulance Transport**

3.1a Do you transport patients to or from your facility?

( ) Yes, specify the frequency per month: ……….. ( ) No

3.1b What type of transport? __ambulance __helicopter __fixed wing __ boat __ other:.........

3.2 How far can your ambulance travel to pick up a patient?

Please specify distance…………………(km) and location(s)…………………………

3.3 If patients were 5km, 10 kms, 25 kms, and 50 km away approximately how long would it take to reach them with an ambulance and bring them back to your facility?

| **Distance** | **Amount of time (min)** | | **Actual pick-up site** |
| --- | --- | --- | --- |
| 5 kms |  | |  |
| 10 kms |  | |  |
| 25 kms |  | |  |
| 50 kms |  |  | |

3.4 How often does you EMS team transport patients using a ground ambulance?

………………a day ………………a month ………………a year

Provide the same type of data if you use helicopter, fixed wing, or boat ambulances (circle)

3.5 Does your hospital provide local patients with a pick-up using an ambulance in case of emergencies or natural disasters (e.g., floods, earthquakes, storms)?

( ) Yes ( ) No

3.6 Do you transport critically ill patients to other hospitals?

( ) Yes, specify transport distance ____ (km) and referral site _______ ( ) No

If yes, why?……………………………………………………………………………

Describe the type of transport used……………………………………………………

3.7 If it is necessary to transfer patients to other hospitals, where do you transfer them and how long does it take?

| **Name of referral hospital** | **Amount of time (min)** |
| --- | --- |
|  |  |
|  |  |
|  |  |
|  |  |
|  |  |

3.8 Identify the community hospital closest to your hospital

- Specify the distance…………………km.
- Specify the travel time……………….min.

3.9 Identify the referral (or regional) hospital closest to your hospital

- Specify the distance…………………km.
- Specify the travel time……………….min.

3.10 Please specify the number of primary care sites, primary care clinics, health promoting hospitals (HPH), POC sites, or primary care units (PCUs) that are your hospital’s responsibility:

| **Name** | **Distance**  **(km)** | | **Travel time**  **(min)** | | **Personnel** | | | | |
| --- | --- | --- | --- | --- | --- | --- | --- | --- | --- |
|  |  |  |  |  | **Physicians** | | **Working hours** | **Nurses** | **Working hours** |
| 1. |  | |  | |  | |  |  |  |
| 2. |  | |  | |  | |  |  |  |
| 3. |  | |  | |  | |  |  |  |
| 4. |  | |  | |  | |  |  |  |
| 5. |  | |  | |  | |  |  |  |
| 6. |  | |  | |  | |  |  |  |
| 7. | |  | |  | |  |  |  |  |
| 8. | |  | |  | |  |  |  |  |
| 9. | |  | |  | |  |  |  |  |
| 10. | |  | |  | |  |  |  |  |

3.11 Now, describe ambulance sites covered from your hospital. State distances, transport times, and destinations.

**Site (pick up) Distance (km) Transport time (min) Destination**

1. _____________ _____________ _____________ __________

2.______________ _____________ _____________ __________

3. _____________ _____________ _ ____________ __________

4. _____________ _____________ _ ____________ __________

5. _____________ _____________ _ ____________ __________

6. _____________ _____________ ____________ __________

7.______________ _____________ ____________ __________

8. _____________ _____________ _ ____________ __________

9. _____________ _____________ _ ____________ __________

10. _____________ _____________ _ ____________ __________

**• POC Testing on Ambulances**

3.12a Specify the POC tests (devices) that are performed (used) in your ambulances and the healthcare providers who perform the testing:...................................................................................

............................................................................................................................................................

3.12b Please rank the tests (devices) listed above in order of the frequency of use in ambulances:

1 (most often) __________ 2 __________ 3 __________ 4 __________ 5 __________

6 _________ 7 _________ 8 __________ 9 __________ 10 (least often) ___________

3.12c Specify the POC tests that your ambulance staff do NOT perform, but you would like to perform in the future:

| **Ambulance Type** | **POC Test(s)** |
| --- | --- |
| Ground |  |
| Helicopter |  |
| Fixed-wing airplane |  |
| Boat |  |
| Other |  |

**• Cardiac Events (e.g., Acute Myocardial Infarction, AMI)**

3.13 Are there health facilities with which you interact for cardiac care? Please fill out the details below. [CH, community hospital; RH, regional hospital]

| **Name** | **Type (CH, RH, other)** | **Distance**  **(km)** | **Time**  **(min)** |
| --- | --- | --- | --- |
|  |  |  |  |
|  |  |  |  |
|  |  |  |  |

3.14 What are the top five facilities and/or technologies you would like to have for cardiac diagnostic support?

| **No** | **Describe** |
| --- | --- |
| 1 |  |
| 2 |  |
| 3 |  |
| 4 |  |
| 5 |  |

3.15 Do you have cardiac care specialists, such as interventional cardiologists?

If so, please describe:……………………………………………..

3.16 Which of the following diagnostic tests do you use to support the diagnosis of acute myocardial infarction (AMI)? (Check all that apply)

POC? Where?___________

( ) High sensitivity cardiac troponin I (hs-cTnI) _____ ___________

( ) High sensitivity cardiac troponin T (hs-cTnT) _____ ___________

( ) Conventional cTnI _____ ___________

( ) Conventional cTnT _____ ___________

( ) Qualitative cTnI _____ ___________

( ) Qualitative cTnT _____ ___________

( ) Creatine kinase myocardial band (CK-MB) _____ ___________

( ) Myoglobin _____ ___________

( ) Other cardiac biomarker(s): _____________ _____ ___________

Circle each of the above that are available at you facility, that is, performed on site.

Indicate which of the cardiac biomarkers are available as POC testing and where performed.

3.17 If you obtain a positive diagnosis of AMI, can you rapid provide rapid interventional care?

( ) Yes, response time:……………… type:………………. ( ) No

- If not, where would the patient travel for care? ______________________________
- What is the travel distance (km)?_____
- What is the travel time (min)?_____
- **What is the total delay in the time to interventional care? __________________(min)**
- **What type of vehicle is used for transport (e.g., ambulance)?___________________**

3.18 What are the next steps if you have a positive diagnosis for the following diseases/ conditions?

| **Diseases/ conditions** | **Next steps** |
| --- | --- |
| Acute Myocardial Infarction |  |
| Acute trauma |  |
| Acute surgeries |  |
| Diabetes Mellitus (Diabetes Ketoacidosis) |  |
| Hypertension |  |
| Stroke |  |
| Asthma |  |
| Sepsis |  |
| Respiratory symptoms |  |
| Digestive symptoms |  |
| Disorders of urethra & urinary tract |  |
| Infectious diseases |  |
| HIV/ sexual transmitted diseases |  |
| COVID-19 |  |
| Other(s)………………………………………………. |  |

3.19 If the next steps are to transfer to another hospital for referral care, to which hospital(s) do you transfer, how long does it take, and how far away is it?

| **Diseases/ conditions** | **Referral hospital** | **Amount of time (min)** | **Distance (km)** |
| --- | --- | --- | --- |
| Acute Myocardial Infarction |  |  |  |
| Acute trauma |  |  |  |
| Acute surgeries |  |  |  |
| Diabetes Mellitus (Diabetes Ketoacidosis) |  |  |  |
| Hypertension |  |  |  |
| Stroke |  |  |  |
| Asthma |  |  |  |
| Sepsis |  |  |  |
| Respiratory symptoms |  |  |  |
| Digestive symptoms |  |  |  |
| Disorders of urethra & urinary tract |  |  |  |
| Infectious diseases |  |  |  |
| HIV/ sexual transmitted diseases |  |  |  |
| COVID-19 |  |  |  |
| Other(s)………………………………………………. |  |  |  |

- **Diabetes Mellitus**

3.20 Where is glucose screening test performed?

( ) Home ( ) Local community ( ) Closest community hospital ฀

( ) Health promoting hospital or primary care unit ( ) Other

3.21 Who does glucose screening test?

( ) Physician ฀ ( ) Nurse ฀ ( ) Public health ฀ staff ( ) Patient

( ) Other, specify

3.22 What type of specimen is used for glucose testing?

( ) Capillary Blood ฀ ( ) Plasma ฀ ( ) Serum ฀ ( ) Other

3.23 Who interprets the result of glucose testing?

( ) Physician ฀ ( ) Nurse ฀ ( ) Public health ฀ staff ( ) Patient

( ) Other

3.24 What cut-offs in mg/dL do you use for prediabetes?

From……… mmol/L to………… mmol/L

3.25 What cut-offs in mg/dL do you use for diabetes?

From……… mmol/L to………… mmol/L

3.26 Is there a diabetic screening program in a community?

( ) Yes ฀ ( ) No

- If so, where is the screening test done?

฀ ( ) Local community ฀ ( ) Closest community hospital ฀

( ) Health promoting hospital ( ) Other

3.27 Do patients use glucose meters to perform self-monitoring of blood glucose level?

( ) Yes ฀ ( ) No

If so, who pays for patients’ glucose meters? Please specify……………………………

3.28 If the patient has complications (e.g., kidney failure, retinopathy), where is the patient treated? Please specify……………………………

3.29 For each of these conditions what diagnostic tests do you perform? Please fill in the tests for the following diseases/ conditions

| **Diseases/ conditions** | **Diagnostic test** |
| --- | --- |
| Acute Myocardial Infarction |  |
| Acute trauma |  |
| Acute surgeries |  |
| Diabetes Mellitus (Diabetes Ketoacidosis) |  |
| Hypertension |  |
| Stroke |  |
| Asthma |  |
| Sepsis |  |
| Respiratory symptoms |  |
| Digestive symptoms |  |
| Disorders of urethra & urinary tract |  |
| Infectious diseases |  |
| HIV/ sexual transmitted diseases |  |
| COVID-19 |  |
| Other(s)………………………………………………. |  |

3.30 What hospital would you find this type of diagnostic testing support?

| **Diseases/ conditions** | **Name of hospital** |
| --- | --- |
| Acute Myocardial Infarction |  |
| Acute trauma |  |
| Acute surgeries |  |
| Diabetes Mellitus (Diabetes Ketoacidosis) |  |
| Hypertension |  |
| Stroke |  |
| Asthma |  |
| Sepsis |  |
| Respiratory symptoms |  |
| Digestive symptoms |  |
| Disorders of urethra & urinary tract |  |
| Infectious diseases |  |
| HIV/ sexual transmitted diseases |  |
| Other(s)………………………………………………. |  |

3.31 What type of POC devices or test kits do you currently have for the following?

| **Diseases/ conditions** | **POC device/ test kit** |
| --- | --- |
| Acute Myocardial Infarction |  |
| Acute trauma |  |
| Acute surgeries |  |
| Diabetes Mellitus (Diabetes Ketoacidosis) |  |
| Hypertension |  |
| Stroke |  |
| Asthma |  |
| Sepsis |  |
| Respiratory symptoms |  |
| Digestive symptoms |  |
| Disorders of urethra & urinary tract |  |
| Infectious diseases |  |
| HIV/ sexual transmitted diseases |  |
| Other(s)………………………………………………. |  |

3.32 In your health network, if your hospital lacks important diagnostic tests during emergencies or natural disasters where do you normally seek for them?

Please specify…………………………………………………………………….

- **Specialized Care**

3.33 For which health facilities does your hospital support if serving as a referral hospital?

Please specify…………………………………………………………………………..

………………………………………………………………………………………….

………………………………………………………………………………………….

3.34 From which health facilities does your hospital need to seek specialized healthcare?

| **Specialized healthcare** | **Health facility** |
| --- | --- |
|  |  |
|  |  |
|  |  |
|  |  |
|  |  |

3.35 In your health network, how would you improve access to care for cardiac arrest patients?

Please explain……………………………………………………………………………

………………………………………………………………………………………………

**SECTION IV. CLINICAL LABORATORY**

- **Service Hours**

4.1 Does your facility have a clinical laboratory? Yes ___ No ___

If yes, please provide the laboratory’s service hours below—

Working days Time:……………….To………………..

Weekends/holidays Time:……………….To………………..

Is on-call service available?฀ ( ) Yes ( ) No

4.2 Please list and rank twenty diagnostic tests that you need the most to care for your patients (1, most important; 20, least important):

*Most Important* 1 __________ 2 __________ 3 __________ 4 __________ 5 __________

6 __________ 7 __________ 8 __________ 9 __________ 10 _________

11 _________ 12 _________ 13 _________ 14 __________ 15 _________

16 _________ 17 _________ 18 _________ 19 __________ 20 _________ *Least*

4.3 Please rank the following laboratory sections in order of importance (1, highest):

_____ Chemistry _____Molecular diagnostics

_____ Hematology _____ Cytology

_____ Microbiology _____ Noninvasive monitoring (pulse oximeter, oxygen saturation monitoring)

_____ Immunology _____ Other (write in the section name): ____________

_____ Microscopy _____ Other (write in): __________________________

_____ Blood Bank _____ Other (write in): __________________________

_____ COVID-19

- **Number of Patients, Locations, and Healthcare Access**

4.4 How many patients do you see a day/ month/ year?

………………per day ………………per month ………………per year

4.5 Where are most of them located in our community?…………………………………..

4.6 How do most of patients get to your facility?

Please specify the following:

………. % Walking

………. % Motorcycles

………. % Automobiles

………. % Public transportation

………. % Ambulances

………. % Helicopter

4.7 Please provide the estimated frequencies of visits for the following diseases/ conditions

| **Diseases/ conditions** | **Estimated frequency of visit** |
| --- | --- |
| Acute Myocardial Infarction |  |
| Acute trauma |  |
| Acute surgeries |  |
| Diabetes Mellitus (Diabetes Ketoacidosis) |  |
| Hypertension |  |
| Stroke |  |
| Asthma |  |
| Sepsis |  |
| Respiratory symptoms |  |
| Digestive symptoms |  |
| Disorders of urethra & urinary tract |  |
| Infectious diseases |  |
| HIV/ sexual transmitted diseases |  |
| COVID-19 |  |
| Other(s)………………………………………………. |  |

- **Oxygen Saturation Monitoring (Pulse Oximetry)**

4.8 Are there any oxygen saturation monitors (pulse oximeters)?

฀ ( ) Yes ฀ ( ) No

If **yes**, identify the number of oxygen saturation monitors ……………………..

4.9 Identify the number of oxygen saturation monitors available in each department:

ER .............................

OR.................................

ICU.............................. .

NICU............................

CCU............................. .

LR................................ .

Ward............................ .

Ambulance....................

Other.............................

- **Critical Care**

4.10 Is blood gas testing performed in the laboratory?

฀ ( ) Yes ฀ ( ) No

If so, which of the following measurements are performed in the laboratory?

pO_2_ ฀ ( ) Yes ฀ ( ) No

pCO_2_ ฀ ( ) Yes ฀ ( ) No

pH ( ) Yes ฀ ( ) No

HCO_3_ ฀ ( ) Yes ฀ ( ) No

4.11 Are electrolytes determined in the laboratory?

฀ ( ) Yes ( ) No

- If so, please specify those electrolytes……………………………………………………

4.12 Who performs the following tests?

- Blood gases ฀ ( ) MD ฀ ( ) Nurse ฀ ( ) Lab staff ฀ ( ) Other…………………
- Electrolytes ( ) MD ฀ ( ) Nurse ฀( ) Lab staff ฀ ( ) Other…………………
- Ionized calcium ( ) MD ฀ ( ) Nurse ฀( ) Lab staff ฀ ( ) Other…………………
- O_2_ ฀( ) MD ฀ ( ) Nurse ฀ ( ) Lab staff ฀ ( ) Other…………………
- pCO_2_ ฀( ) MD ฀ ( ) Nurse ฀ ( ) Lab staff ฀ ( ) Other…………………
- pH ฀( ) MD ฀ ( ) Nurse ฀ ( ) Lab staff ฀ ( ) Other…………………
- HCO_3_ ( ) MD ( ) Nurse ( ) Lab staff ( ) Other…………………

4.13 Does the laboratory perform bloodstream pathogen cultures? ฀

( ) Yes ( ) No

4.14 Which and where of the following tests are performed?

- Hepatitis A ฀ ( ) Yes ( ) No Where…………………
- Hepatitis B (HBsAg) ( ) Yes ( ) No Where…………………
- Hepatitis B (HB Ab) ฀ ( ) Yes ( ) No Where…………………
- Influenza A ( ) Yes ( ) No Where…………………
- Influenza B ( ) Yes ( ) No Where…………………
- H1N1 ( ) Yes ( ) No Where…………………
- H7N9 ( ) Yes ( ) No Where…………………
- Mers-CoV ( ) Yes ( ) No Where…………………
- COVID-19 ( ) Yes ( ) No Where…………………
- HIV (AIDS) ฀ ( ) Yes ( ) No Where…………………
- Strep throat ( ) Yes ( ) No Where…………………
- Blood culture ฀ ( ) Yes ( ) No Where…………………
- Growing on culture plate ( ) Yes ( ) No Where…………………

4.15 What type of tests do you offer for COVID-19 (e.g., antigen tests, LAMP, PCR, antibody)?

- **Cardiovascular disease**
  1. Which of the following tests are performed by the on-site laboratory? (Check all that apply)

( ) High sensitivity cardiac troponin I (hs-cTnI)

( ) High sensitivity cardiac troponin T (hs-cTnT)

( ) Conventional cTnI

( ) Conventional cTnT

( ) Qualitative cTnI

( ) Qualitative cTnT

( ) Creatine kinase (CK)

( ) Creatine kinase myocardial band (CK-MB)

( ) Myoglobin

( ) Lactate dehydrogenase (LDH)

( ) Aspartate aminotransferase (AST)

( ) B-type natriuretic peptide (BNP)

( ) N-terminal pro-BNP (NT-proBNP)

( ) Other biomarker(s): _____________________________________________________

- **Hematology**

4.17 Is there a complete blood count testing?

( ) Yes ( ) No

4.18 Is there a coagulation testing?

( ) Yes ( ) No

- If so, what types of tests are performed in the laboratory?

( ) PT ( ) aPTT ฀ ( ) PT and aPTT ( ) Other……………………….

4.19 Do the nurses perform any laboratory diagnostic tests the same as laboratory staff do?

( ) Yes ฀ ( ) No

- If so, please specify the laboratory tests the nurses can perform

฀ ( ) Hematology, specify…………………………………………………

฀ ( ) Serology, specify……………………………………………………

฀ ( ) Microbiology, specify……………………………………………….

฀ ( ) Chemistry, specify…………………………………………………..

฀ ( ) Urinalysis, specify…………………………………………………..

( ) Other, specify………………………………………………………

4.20 What any new instruments or tests does your laboratory still need?

………………………………………………………………………………………………………

4.21 What test(s) does your laboratory need to send to outside laboratory or referral laboratory?

………………………………………………………………………………………………………

4.22 Please specify the name and location of outside laboratory or referral laboratory

………………………………………………………………………………………………………

4.23 how long does it take to receive the testing results from the outside lab or referral lab (Identify turnaround time)

………………………………………………………………………………………………………

4.24 What types of tests are performed at health promoting hospitals?

฀ ( ) Glucose Test ฀ ( ) Pregnancy Test ฀( ) Urine Test

฀ ( ) Protein Test ฀( ) Hematocrit ฀ ( ) Other

4.25 What new instruments or tests would help improve patients’ health outcomes at health promoting hospitals?

4.26 Who directs the diagnosis testing at health promoting hospitals?

฀( ) MD ( ) Nurse ( ) Lab staff ( ) Public health staff ( ) Other

4.27 Who is responsible for doing quality control of the diagnostic testing at health promoting hospitals?

( ) MD ( ) Nurse ( ) Lab staff ( ) Public health staff ( ) Other

**SECTION V. MEDICAL PROBLEM SOLVING IN THE COMMUNITY**

5.1 Please list and rank the most common medical problems in your community (1, top; 10, least important)

1…………………………………(top) 6. ……………………………………….

2………………………………………. 7. ……………………………………….

3………………………………………. 8. ……………………………………….

4………………………………………. 9. ……………………………………….

5………………………………………. 10. ……………………………………….

5.2 Which medical problems are neglected by patients and/.or healthcare providers?

Please specify………………………………………………………………………………

5.3 What medical problems influence peoples’ working performance the most?

Please specify………………………………………………………………………………

5.4 In case of emergencies, where do the people turn to receive healthcare delivery?

Please specify………………………………………………………………………………

5.5 What are the most common patient diagnoses that you cannot treat at your own hospital and therefore, must refer to another hospital? List and rank: (1, top; 5, least important)

1……………………………………….(most common)

2……………………………………….

3……………………………………….

4……………………………………….(least common)

5……………………………………….

**• Special COVID-19 Pandemic Questions**

Please fill in the blanks for the following questions and pandemic diagnostic testing—

5.6 Do people under your care do home self-testing for COVID-19?............................................

5.7 What COVID-19 tests are available in your community?.........................................................

5.8 Are COVID-19 tests free (no charge)? Yes__ No__ If not free, what is the cost?.................

5.9 Are mobile vans used for COVID-19 testing in your community?...........................................

5.10 Which COVID-19 tests are used the most (list in order)?.......................................................

5.11 What is the frequency of *false positive test results* you observe?...........................................

5.12 What is the frequency of *false negative test results* that you observe?...................................

5.13 What is the prevalence of COVID-19 in your community?....................................................

**SECTON VI. SUMMARY OF OBSERVATIONS**

Please identify the most important and highest impact improvements that POC testing (POCT) could produce at this site or in its surrounding healthcare small-world network (SWN) based on actual needs assessment evidence obtained during site visits to SWN resources. Separate your observations into a) COVID-19 testing, b) cardiac biomarkers, c) emergency room and ambulance POC testing, d) POC strategic changes, and e) improvements in geospatial care paths (the fastest routes taken by patients when seeking diagnosis and care).

***A) COVID-19*** [rapid antigen tests, molecular diagnostics (e.g., PCR, LAMP), antibody tests] .…………………………………………………………………………………………………………………………………………………………………………………………………………………………………………………………………………………………………………………..

***B) Cardiac Biomarkers*** ………………………………………………………………………………………………………………………………………………………………………………………………………………………………………………………………………………………………………………………

***C) Ambulance POC Tests***

………………………………………………………………………………………………………………………………………………………………………………………………………………………………………………………………………………………………………………………

***D) POC Strategic Changes*** ………………………………………………………………………………………………………………………………………………………………………………………………………………………………………………………………………………………………………………………

***E) Improvements in Geospatial Care Paths***

………………………………………………………………………………………………………………………………………………………………………………………………………………………………………………………………………………………………………………………

***Version 12.5-Revision 1.8***

***November 22, 2023***
